# Effectiveness of simultaneous bilateral visual diaphragm biofeedback under low back pain: influence of age and sex

**DOI:** 10.3389/fphys.2024.1407594

**Published:** 2024-07-09

**Authors:** Nerea Molina-Hernández, David Rodríguez-Sanz, José López Chicharro, Ricardo Becerro-de-Bengoa-Vallejo, Marta Elena Losa-Iglesias, Davinia Vicente-Campos, Daniel Marugán-Rubio, Samuel Eloy Gutiérrez-Torre, César Calvo-Lobo

**Affiliations:** ^1^ Faculty of Nursing, Physiotherapy and Podiatry, Universidad Complutense de Madrid, Madrid, Spain; ^2^ Grupo FEBIO, Universidad Complutense de Madrid, Madrid, Spain; ^3^ Faculty of Health Sciences, Universidad Rey Juan Carlos, Madrid, Spain; ^4^ Faculty of Health Sciences, Universidad Francisco de Vitoria, Madrid, Spain; ^5^ Centro Superior de Estudios Universitarios La Salle, Madrid, Spain

**Keywords:** biofeedback, breathing exercises, diaphragm, low back pain, ultrasonography

## Abstract

**Introduction:** The aim of the present study was to determine the effectiveness of simultaneous bilateral visual diaphragm biofeedback (BFB) from ultrasonography in conjunction with inspiratory muscle training (IMT) on diaphragmatic thickness during normal breathing and respiratory and clinical outcomes in patients with non-specific low back pain (NSLBP) and determine the influence of age and sex.

**Methods:** A single-blind randomized clinical trial was carried out (NCT04582812). A total sample of 96 patients with NSLBP was recruited and randomized by sex-based stratification into IMT (*n* = 48) and BFB + IMT (*n* = 48) interventions over 8 weeks. Bilateral diaphragmatic thickness at maximum inspiration (T^ins^) and expiration (T^exp^), respiratory pressures, lung function, pain intensity, bilateral pressure pain threshold (PPT), disability, and quality of life were measured at baseline and after 8 weeks.

**Results:** The BFB + IMT group showed significant differences (*p* < 0.05) with increased left hemidiaphragm thickness at T^ins^ and T^ins-exp^ (*d* = 0.38–053), and right and left PPT (*d* = 0.71–0.74) *versus* the IMT group. The interaction with sex was statistically significant (*p* = 0.007; F_(1,81)_ = 7.756; η_p_^2^ = 0.087) and higher left hemidiaphragm thickness at T^ins^ was predicted by the BFB + IMT group (*R* ^2^ = 0.099; β = 0.050; F_(1,82)_ = 8.997; *p* = 0.004) and male sex (*R* ^2^ = 0.079; β = 0.045; F_(1,81)_ = 7.756;*p* = 0.007). Furthermore, greater left hemidiaphragm thickness at T^ins-exp^ was predicted by younger age (*R* ^2^ = 0.052; β = −0.001; F_(1,82)_ = 4.540; *p* = 0.036).

**Discussion:** The simultaneous bilateral visual diaphragm biofeedback by ultrasonography in conjunction with IMT was effective in both increasing the left diaphragmatic thickness during inspiration, which was positively influenced and predicted by male sex and younger age, and increasing the bilateral PPT of the paraspinal muscles in patients with NSLBP.

## 1 Introduction

Non-specific low back pain (NSLBP) has been stated as the most common musculoskeletal condition, providing an 80% rate of estimated cumulative incidence among the population throughout their working lives, and is associated with greater disability and reduced quality of life and other biopsychosocial factors (Maher et al., 2017). Sex and age distribution were linked to different prognoses, pain sensitivity, and response to NSLBP management (Lobo et al., 2017; Alhowimel et al., 2018; Ferrer-Peña et al., 2018; Schilter et al., 2024). The point, year, and life prevalence of NSLBP reached ratios of up to 67%, 94%, and 84%, respectively (Farahbakhsh et al., 2018). In Europe, the direct costs secondary to this musculoskeletal disorder exceed 7,000 euros per person per year (Juniper et al., 2009).

This condition has been described as a complex disorder including factors from various dimensions, such as movement, pain sensitivity, psychological aspects, and work conditions, which may influence both central and peripheral nociceptive processes (Rabey et al., 2019). Growing evidence suggested that current applied clinical practice was discordant with respect to contemporary evidence, exacerbating the psychological and fear–avoidance beliefs as well as the lack of response to different interventions (O’Sullivan et al., 2016). According to the multidimensional and complex approach necessary to manage the NSLBP, novel individualized and patient-centered care treatments should be applied to improve their effectiveness (Rabey et al., 2019).

Recently, the presence of NSLBP has been linked to respiratory disorders (Beeckmans et al., 2016). Specifically, patients with NSLBP presented a reduced bilateral diaphragm muscle thickness during normal breathing, and diaphragm respiratory training could play a key role in the rehabilitation of patients with this condition (Calvo-Lobo et al., 2019; Vicente-Campos et al., 2021). Consequently, maximum respiratory pressures and spirometry parameters in NSLBP patients suggested that this disorder may be associated with respiratory muscle weakness and worse pulmonary function in accordance with the abnormal position and postural control of the diaphragm (Kolar et al., 2012; Mohan MPT et al., 2018; Uddin and Vaish, 2023).

Among the different treatments to improve respiratory and clinical findings under NSLBP (Usman et al., 2023), inspiratory muscle training (IMT) was considered an effective intervention to restore respiratory function, stabilize core muscles, and improve postural control and pain sensitivity (Gholami Borujeni and Yalfani, 2019; Ahmadnezhad et al., 2020). Indeed, high-intensity IMT over 8 weeks produced improvements in postural control, respiratory muscle strength, and pain severity in patients with NSLBP (Janssens et al., 2015). Furthermore, a 6-week training program using unilateral visual biofeedback of each hemidiaphragm separately during normal breathing by ultrasonography in conjunction with the aforementioned IMT protocol improved lung function under NSLBP (Marugán-Rubio et al., 2021; 2022).

The inspiratory muscle activity of both the right and left hemidiaphragms seemed to be bilaterally and simultaneously performed (Boussuges et al., 2009). Therefore, simultaneous and bilateral ultrasound diaphragm visual biofeedback with a bilateral thoracic orthosis device was reliable and could improve the diaphragm reeducation during normal breathing activity (Molina-Hernández et al., 2023). Almost 40 years ago, the contractile properties of the human diaphragm were studied under simultaneous bilateral contraction, and the unilateral contraction of each hemidiaphragm separately led to distortion and non-normal changes in diaphragm geometry (Bellemare et al., 1986). Moreover, sex-based and aged-based fatigability of the diaphragm muscle may influence exercise performance (Fogarty et al., 2019; Andrew Harry Ramsook BPHE, 2021). We hypothesized that the simultaneous bilateral visual biofeedback about the diaphragm by ultrasonography, in addition to IMT, could improve the respiratory and clinical findings under NSLBP, influenced by age and sex characteristics. First, the main aim of the present study was to determine the effectiveness of an intervention using simultaneous bilateral visual diaphragm biofeedback by ultrasonography on diaphragmatic thickness during normal breathing added to IMT *versus* the isolated application of IMT in patients with NSLBP. Second, the secondary purposes of this study were to establish the effectiveness of this simultaneous bilateral visual diaphragm biofeedback intervention plus IMT with respect to isolated IMT on other respiratory outcomes such as respiratory muscle strength by maximum respiratory pressures and lung function by spirometry, as well as clinical outcomes including pain intensity, pressure pain threshold (PPT), disability, and quality of life in patients with NSLBP. Lastly, the additional aims of this study were to determine the influence of age and sex on the aforementioned outcomes.

## 2 Materials and methods

### 2.1 Trial design and registry

A single-blinded (evaluator), parallel-groups, randomized clinical trial was prospectively registered by the number clinical trial NCT04582812 at ClinicalTrials.gov and performed from 30 November 2022 to 19 February 2024, following the Consolidated Standards of Reporting Trials (CONSORT 2010) criteria (Schulz et al., 2010).

### 2.2 Ethical aspects

The Helsinki Declaration and all ethical requirements regarding human experimentation were respected (Holt, 2014). The study was approved on 18 November 2020 by the ethics committee of the San Carlos Clinical Hospital (Madrid, Spain) with the approval code 20.655-E_BS. All patients included in the present study received the information sheet and signed the informed consent form.

### 2.3 Research project

This research study was supported and funding by the Spanish Ministry of Science, Innovation and Universities and the State Agency for investigation of the national government regarding the Call for Innovation, Development and Research (I + D + i Projects) in 2020, according to the framework of the State Program for Knowledge Generation for Scientific and Technological Strengthening of the I + D + i System as well as I + D + i oriented to Society Challenges with the grant number PID 2020-117162RA-I00 funded by MICIU/AEI/10.13039/501100011033.

### 2.4 Patent registry

A patent registry was performed for a utility model in the Spanish Patent and Trademark Office with application number U202200045, publication number ES1288519, and issue date of 30 March 2022. This bilateral thoracic orthosis, including both the right and left holding devices for two ultrasound probes, showed reliability from good to excellent and adequate repeatability for the simultaneous thickness measurement of both hemidiaphragms bilaterally during normal breathing. The use of this device was previously recommended for simultaneous breathing reeducation of both the right and left hemidiaphragms by ultrasonography visual biofeedback (Molina-Hernández et al., 2023).

### 2.5 Calculation for sample size

The sample size calculation for this study was carried out using the difference for two independent groups by the 3.1.9.2 version of the G*Power program (G*Power^©^; Dusseldorf University; Germany) (Faul et al., 2007), considering the difference in the thickness of the left hemidiaphragm during inspiration as the main outcome, given that this measurement was associated with muscular alterations in the lumbar region, as this hemidiaphragm was claimed to play a key role in postural function (Celli, 1989; Hruska, 1997; Terada et al., 2016), and a moderate effect size with a Cohen’s *d* of 0.63 necessary to normalize the difference in diaphragmatic thickness of patients with NSLBP with respect to healthy subjects (Calvo-Lobo et al., 2019), using a two-tailed hypothesis, a probability of error α of 0.05, a power of 0.80 (1-β probability of error), and a randomization rate of 1 (N_2_/N_1_). According to these parameters, a sample size of 82 patients with NSLBP was necessary to achieve an actual power of 0.804, divided into two groups of 41 patients in each intervention group. Considering a 15% possible loss to follow-up, 96 patients with NSLBP were recruited for the total sample size, including 48 patients with NSLBP per group.

### 2.6 Recruitment and sampling

A total sample of 96 patients with NSLBP was recruited by announcements from the different health sciences faculties of the Complutense University of Madrid (Madrid, Spain) from 30 November 2022 to 23 March 2023. A sex-based stratified random sampling method was developed to recruit 48 men and 48 women in order to determine the influence of sex on the outcomes that showed effectiveness after comparing both interventions. Both treatment groups were pair-matched by sex, including 24 men and 24 women for each intervention group (Berndt, 2020).

### 2.7 Sample characteristics

Inclusion criteria comprised patients, including students, professors, or persons coming from outside the university, with bilateral NSLBP medical diagnosis with duration for more than 6 weeks and a pain location mainly between the bi-iliac line and the subcostal line as well as a positive active straight leg raise test bilaterally, aged between 18 and 65 years (Patel et al., 2016; Calvo-Lobo et al., 2019). Exclusion criteria comprised congenital lumbar disorders, rheumatic or neuromuscular disorders, body mass index (BMI) greater than 31 kg/m^2^, previous diagnosis of respiratory or neurological pathology, previous surgery and lower limb pathology (including fractures, sprains, or joint instability), skin disorders, pregnancy, inability to follow instructions during the study, and presence of hyperventilation syndrome as assessed by the Nijmegen test indicated by a score equal to or greater than 24 points (Paris-Alemany et al., 2018; Calvo-Lobo et al., 2019; Marugán-Rubio et al., 2021).

### 2.8 Randomization, procedure, and blinding

A simple randomization process was performed by applying based-sex stratification in order to allocate the 96 patients with NSLBP into both intervention groups (including 24 men and 24 women for each group) using the 4.1 version of the EPIDAT program (Xunta de Galicia; Conselleria de Sanidade; Galicia; Spain) (Berndt, 2020).

According to this randomization process, 48 patients (24 men and 24 women) were assigned to high-intensity inspiratory muscle self-training intervention for 8 weeks (Janssens et al., 2015; Marugán-Rubio et al., 2022) (IMT group) and 48 patients (24 men and 24 women) were also assigned to the same IMT for 8 weeks plus simultaneous bilateral visual biofeedback of the diaphragm by ultrasound imaging for 6 weeks (BFB + IMT group) (Hides et al., 2008; Janssens et al., 2015; Marugán-Rubio et al., 2022; Molina-Hernández et al., 2023). Therapists with more than 4 years of experience in IMT and BFB applied both interventions. Outcome measurements were performed at baseline and after 8 weeks of intervention by a physician together with other evaluators experienced in ultrasound imaging of the diaphragm who were blinded to the intervention group allocation using numerical coding (Marugán-Rubio et al., 2022).

### 2.9 Intervention groups

**FIGURE 1:**
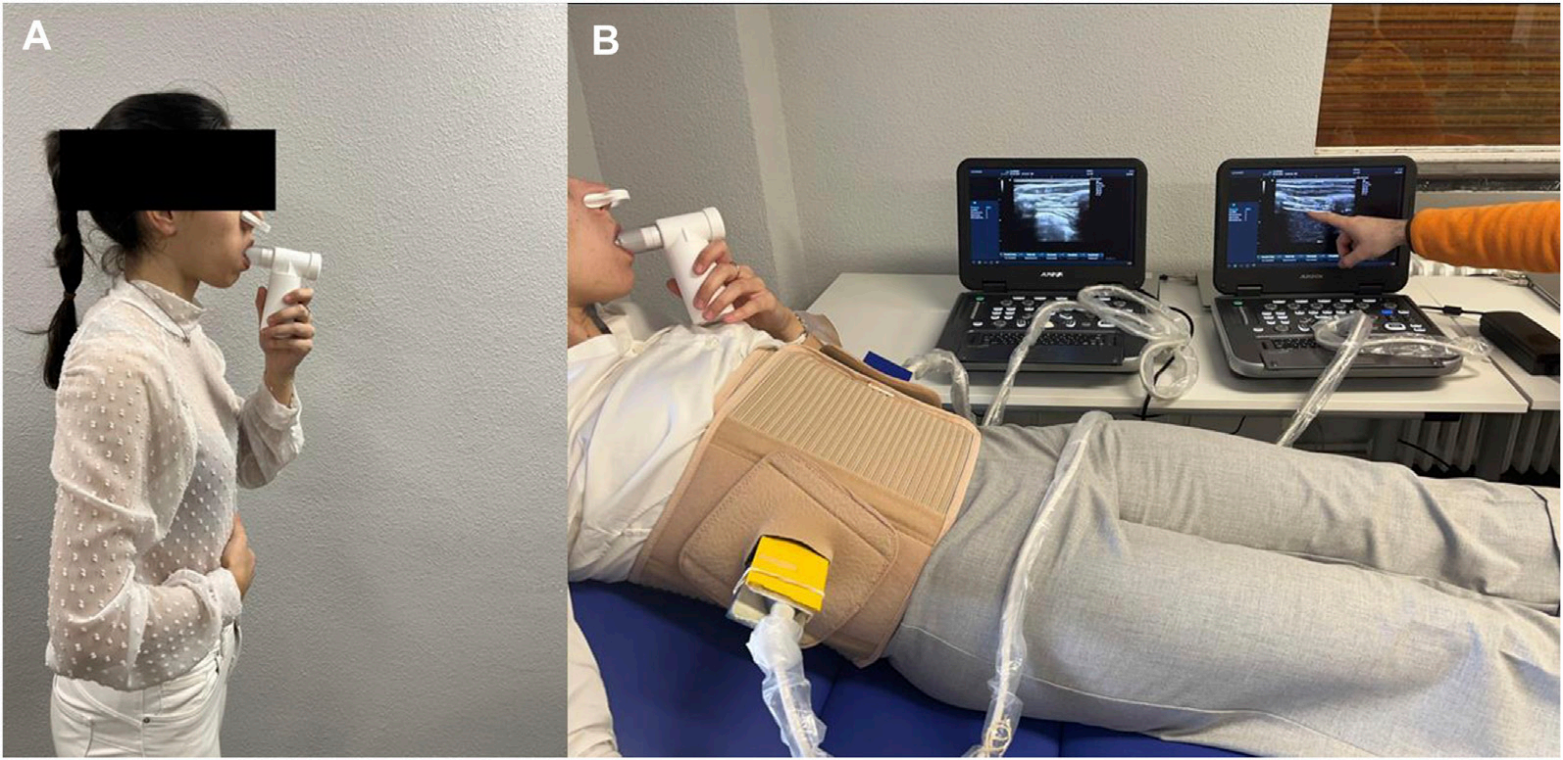
**(A)** High-intensity inspiratory muscle self-training (IMT) using the POWERbreathe device from Medic. **(B)** Simultaneous bilateral ultrasonography visual biofeedback within the proposed orthosis device using two holding devices to fix the right and left ultrasound probes perpendicular to the last intercostal space following the right and left mid-axillary lines.

The IMT group received only high-intensity inspiratory muscle self-training for 8 weeks. Patients were instructed to breathe using a mouthpiece (POWERbreathe, Medic, HaB International Ltd., Warwickshire, United Kingdom) that occluded their nose in a standing position and produced a negative pressure according to 60% of their maximum inspiratory pressure (MIP) using an inspiratory valve that resisted each inspiration effort (Figure 1A). Patients performed 30 breaths twice per day, 7 days per week, with a rate of 15 breaths per minute and applying a duty cycle of 0.5. Furthermore, all NSLBP participants were trained to apply mainly diaphragmatic breathing, named the “bucket-handle” motion, rather than thoracic breathing, named the “pump arm” motion, through the provision of tactile and verbal signals (Janssens et al., 2015; Marugán-Rubio et al., 2022).

The BFB + IMT group received the same high-intensity IMT for 8 weeks plus simultaneous bilateral visual biofeedback of the diaphragm by ultrasound imaging for 6 weeks using the proposed bilateral thoracic orthotic device for reeducation of both hemidiaphragms at the same time during normal breathing (Figure 1B). Patients were instructed in the same way for high-intensity IMT self-training in conjunction with diaphragmatic breathing reeducation using ultrasound visual biofeedback with the proposed thoracic orthosis to facilitate probe fixation and visualization of the ultrasound screen, selectively explaining diaphragmatic thickening during inspiration and correcting paradoxical breathing patterns (Hides et al., 2008; Janssens et al., 2015; Calvo-Lobo et al., 2019; Marugán-Rubio et al., 2022; Molina-Hernández et al., 2023). The same two high-quality ultrasonography tools (E-CUBE i7, Alpinion Medical Systems, Seoul, Korea) and the two linear probes (Broadband Linear type L3_12T; 38.4 mm field of view, 128 elements) with a frequency range from 8.0 to 12.0 MHz and a 45 mm probe foot were used for the measurements and the interventions. Transcostal diaphragmatic thickness was evaluated by B-mode imaging at rest in a supine position with a preset depth of 3 cm, 12 MHz frequency, 64-point gain, 64-point dynamic range, and one focus located at 2 cm depth (Calvo-Lobo et al., 2019; Marugán-Rubio et al., 2022; Molina-Hernández et al., 2023). The registered patent comprised two holding devices that fixed the right and left ultrasound probes to the thoracic orthosis by two bivalve adapters, permitting the insertion and fixation of both ultrasound probe holders without interfering with the patients’ breathing patterns (Marugán-Rubio et al., 2021; Molina-Hernández et al., 2023). This device allowed total thoracic mobility. The addition of ultrasound gel in the spaces below each probe footprint permitted a complete visualization of the last intercostal space. Both the right and left linear ultrasound probes were bilaterally placed perpendicular to the last intercostal space following the right and left mid-axillary lines of the patient, who was located in the supine decubitus (Harper et al., 2013; Molina-Hernández et al., 2023). This bilateral thoracic orthosis presented good to excellent reliability and adequate repeatability with an intraclass correlation coefficient (ICC) of 0.614 to 0.997, a standard error of measurement (SEM) of 0.002 to 0.028 cm, and minimum detectable change (MCD) of 0.006 to 0.079 cm for the bilateral simultaneous thickness evaluation of both hemidiaphragms during normal breathing. The orthosis was previously recommended for simultaneous breathing reeducation of both the right and left hemidiaphragms by ultrasonography visual biofeedback (Molina-Hernández et al., 2023).

### 2.10 Descriptive data

Descriptive data such as sex (dichotomous categorical variable, male or female), age (continuous quantitative variable detailed in years), height (continuous quantitative variable detailed in cm), weight (continuous quantitative variable described in kg), and BMI (continuous quantitative variable detailed in kg/cm^2^ according to the Quetelet index (Garrow, 1986)) were detailed (Hides et al., 2008; Janssens et al., 2015; Calvo-Lobo et al., 2019; Marugán-Rubio et al., 2022; Molina-Hernández et al., 2023). The International Physical Activity Questionnaire (IPAQ), a questionnaire with adequate psychometric properties, was applied to determine the rate of metabolic equivalents per task per minute per week (METs/min/wk; measured as a continuous quantitative variable) and categorized according to the level of physical activity into level I—sedentary (<600 METs/min/wk), level II—moderate (600–1500 METs/min/wk), or level III—vigorous (>1500 METs/min/wk), as a polytomous categorical variable (Gauthier et al., 2009; Marugán-Rubio et al., 2022; Molina-Hernández et al., 2023).

### 2.11 Primary outcome

#### 2.11.1 Ultrasonography diaphragm thickness during normal breathing

Ultrasound measurements were unilaterally performed using the thoracic orthosis device by the fixation of each ultrasound probe to measure right and left diaphragm thickness during normal breathing using a randomized order for the ultrasound evaluation side. These images were coded, saved, and assessed using ImageJ software by blinded evaluators (Calvo-Lobo et al., 2019; Marugán-Rubio et al., 2021; Marugán-Rubio et al., 2022).

Transcostal ultrasonography measurements of the right and left hemidiaphragm thicknesses were carried out in cm during maximum inspiration time (T^ins^) and maximum expiration time (T^exp^), and their thickness difference (T^ins-exp^) was calculated during normal breathing. The same two high-quality ultrasound tools of the visual biofeedback intervention were used for B-mode ultrasound image evaluations (E-CUBE i7, Alpinion Medical Systems, Seoul; Korea). These ultrasonography images were also made using the same two linear probes (L3_12T-type; 34 mm field of view; 128 elements) used in the visual biofeedback intervention, with a frequency from 8.0 MHz to 12.0 MHz and a 45 mm footprint. Diaphragm thickness measurements were performed in a supine position by B-mode ultrasound imaging using a pre-fixed pre-set including depth of 3 cm, frequency 12 MHz, 64 points of gain and dynamic range, and one focus located at a depth of 2 cm (Harper et al., 2013; Calvo-Lobo et al., 2019; Marugán-Rubio et al., 2021; Marugán-Rubio et al., 2022).

**FIGURE 2:**
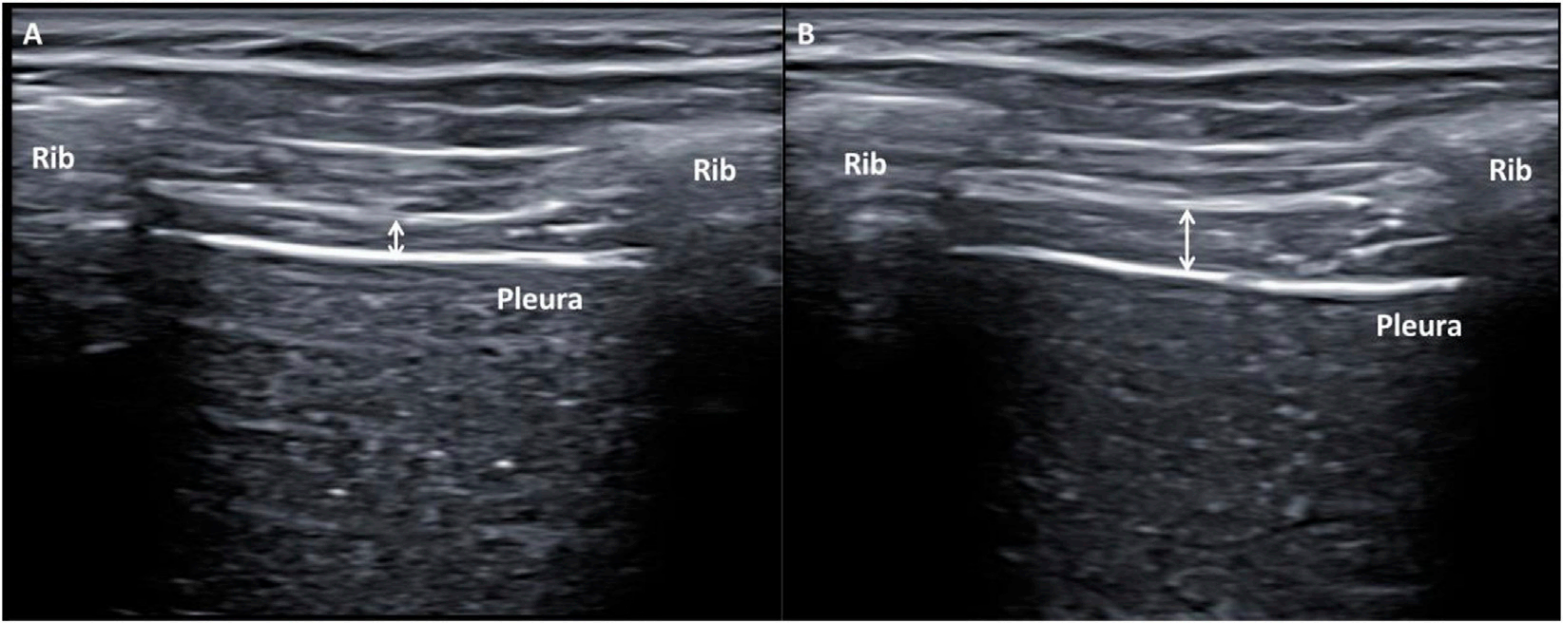
**(B)** Mode ultrasonography imaging of the hemidiaphragm thickness showing the last intercostal space following the mid-axillary line from the 12th rib upper edge to the 11th rib lower edge of the thorax region during normal breathing. **(A)** Hemidiaphragm thickness measurement marked by a white arrow during maximum expiration time (T^exp^). **(B)** Hemidiaphragm thickness measurement marked by a white arrow during maximum inspiration time (T^ins^).

Ultrasonography images were created in gray-scale and converted to digital imaging and communications in medicine (DICOM) format and calibrated through ImageJ software version 2.0 (U.S. National Institutes of Health; Bethesda; Maryland; United States) to measure the thicknesses of the right and left hemidiaphragms. Both linear probes were placed perpendicular to the last intercostal space following the mid-axillary line from the 12th rib upper edge to the 11th rib lower edge of the thorax region, permitting adequate bilateral diaphragm visualization below the hyper-echogenic connective tissue corresponding to intercostal muscles during normal breathing (Figure 2) (Calvo-Lobo et al., 2019; Marugán-Rubio et al., 2021; Marugán-Rubio et al., 2022; Molina-Hernández et al., 2023).

Lastly, measurements were repeated three times to establish the thickness of both the right and left hemidiaphragms at T^ins^, T^esp^, and T^ins-esp^ for each parameter. Thickness measurements for each hemidiaphragm were recorded by placing each electronic caliper inside the two hyper-echogenic lines, which corresponded to the connective tissue around the diaphragm muscle, and measuring the thickness of each hemidiaphragm at the center of the intercostal space. The mean value was calculated from the three repeated measurements. Ultrasound measurements were unilaterally performed using the thoracic orthosis device to fix each ultrasound probe and measure both hemidiaphragm thicknesses during normal breathing because this measurement procedure reduced measurement errors and was better than manual or simultaneous bilateral measurements. The measurement had excellent reliability, showing an ICC of 0.852 to 0.996, an SEM of 0.0002–0.054 cm, and an MDC of 0.002–0.072 cm, avoiding systematic errors of measurement (Marugán-Rubio et al., 2021; Molina-Hernández et al., 2023).

### 2.12 Secondary respiratory outcomes

#### 2.12.1 Respiratory muscle strength

Respiratory muscle strength was measured by the maximum inspiratory pressure (MIP) and maximum expiratory pressure (MEP) through the RP-Check tool (MD Diagnostics Ltd., Chatham, United Kingdom) because the residual volume was in line with the recommendations proposed by the American Thoracic Society and the European Respiratory Society (Graham et al., 2017; 2019). Both MIP and MEP were measured in cmH_2_O in order to compare the absolute values of both intervention groups. The measurement protocol was repeated and performed at least three times or up to two reproducible efforts (within 5% of each other). Intervals of 1 min were applied among these measurements in order to avoid respiratory muscle fatigue in the short term. The highest of two reproducible values was used for the data analysis (Vicente-Campos et al., 2021; Marugán-Rubio et al., 2022). The described procedure showed excellent interrater reliability with an ICC of 0.914 to 0.925 (Cofré et al., 2018).

#### 2.12.2 Lung function

Spirometry respiratory parameters evaluated the airway airflow restriction through the Datospir-600 touch device (SIBEL S.A.U., Barcelona, Spain), including the forced expiratory volume for 1 second (FEV_1_) in L, forced vital capacity (FVC) in L, and the ratio of FEV_1_/FVC in %. These are the most important spirometry parameters that show airway disturbance at a physiological level (Calvo-Lobo et al., 2018; Marugán-Rubio et al., 2022). The displayed values reflected the lung function with *r* correlations of 0.74 with respect to the expansion of the chest wall and good reliability with an ICC of 0.786 to 0.929 (Calvo-Lobo et al., 2018).

### 2.13 Secondary clinical outcomes

#### 2.13.1 Pain intensity

Pain intensity was self-reported by patients considering the average value during the last week at rest using a paper-based visual analog scale (VAS), which comprised a horizontal line of 10 cm where NSLBP patients marked with a pencil the point of pain intensity, from “no pain” at the left side to the “worst pain imaginable” at the right side (Boonstra et al., 2008; Marugán-Rubio et al., 2022). This tool showed adequate reliability and validity within an ICC of 0.88 to measure pain intensity in the last week and an adequate correlation of *r* of 0.76 with disability in patients with musculoskeletal pain (Boonstra et al., 2008; Ferrer-Peña et al., 2018). In addition, the ICC, SEM, and MDC of the VAS were set at 0.97 points, 0.03 cm, and 0.08 cm, respectively, in patients with musculoskeletal disorders (Alghadir et al., 2018).

#### 2.13.2 Pressure pain threshold

Mechanosensitivity was determined by the pressure pain threshold (PPT) over 0–10 kg/cm^2^ through a mechanical algometry tool (Wagner Instruments; Greenwich; CT). This tool measured the paraspinal muscle’s mechanosensitivity with an ICC of 0.91, a coefficient of variation of 10.3%, an SEM of 0.19 kg/cm^2^, and an MDC of 0.54 kg/cm^2^ (Koo et al., 2013). This device showed excellent reliability, sensitivity, and reproducibility to assess the PPT at the center of the paraspinal muscles when applied bilaterally and perpendicular to the L3 spinous process. All measurements were manually carried out by a gradual protocol until the patient mentioned feeling pain. Measurements were repeated three times at the same point with an interval of 30–60 s, calculating the mean of the three repeated measurements for data analysis (Koo et al., 2013; Calvo-Lobo et al., 2017; Marugán-Rubio et al., 2022).

#### 2.13.3 Disability

Disability was self-reported by NSLBP patients using the Spanish Roland–Morris Disability Questionnaire (RMDQ), which showed adequate validity with a Cronbach’s alpha of 0.91 and test–retest reliability with an ICC of 0.87. The questionnaire included 24 items that assessed the daily life limitations linked to NSLBP from 0, indicating “no disability,” to 24 points, reporting “maximum disability” (Kovacs et al., 2002; Calvo-Lobo et al., 2019; Marugán-Rubio et al., 2022). The SEM and MDC were stated at 2.48 and 5.00 points, respectively, in patients with NSLBP (Jenks et al., 2022).

#### 2.13.4 Quality of life

Quality of life was self-reported by patients with NSLBP by applying the 12-item Short Form (SF–12) health questionnaire, measuring the health-related quality of life to evaluate both the physical and mental health domains as well as the total score. It is a valid and reliable tool with a Cronbach α of 0.78 to 0.85 (Vilagut et al., 2008; Marugán-Rubio et al., 2022). Moreover, ICC, SEM, and MDC were set at 0.86, 3.82, and 8.90 points for the physical health domain, while 0.77, 5.92, and 13.80 points were set for the mental health domain in patients with musculoskeletal alterations (Clement et al., 2019).

### 2.14 Statistical analyses

For the main and secondary purposes of this research study, the Statistical Package of Social Sciences (SPSS) version 24.0 software application (IBM; Armonk, NY, IBM Corp.) was used for the data analysis to compare descriptive data and primary and secondary outcomes between both intervention groups. An α error of 0.05 was applied, and *p*-values <0.05 were determined to be statistically significant for a confidence interval (CI) of 95%. Raw data of the present study may be accessed at Data File S1. All analyses were carried out regarding two intervention groups and the difference between two measurement times (baseline before intervention and after 8 weeks of intervention). The Kolmogorov–Smirnov statistical test was applied to determine the distribution normality. This statistical test was recommended in the field of health sciences for sample sizes larger than 30 patients per group (Ghasemi and Zahediasl, 2012). Mean ± standard deviation (SD), lower and upper limits according to 95% CI, and range (minimum and maximum values) were determined for all data. Student’s t-tests were used to compare differences between the two independent groups regarding parametric data using the *p*-value following Levene’s tests of variance equality (*p*-value <0.05 if there were no variance equalities). The differences between the two independent groups regarding non-parametric data were determined by Mann–Whitney *U* tests. Frequencies (n) and percentages (%) were applied to describe all categorical data. The comparison of categorical data among groups was performed by Fisher’s exact tests if dichotomous variables were analyzed or chi-squared (χ^2^) tests if polytomous variables were tested. Moreover, the effect sizes of the comparisons between the two intervention groups for the primary and secondary outcomes were calculated by applying Cohen’s *d* calculated by the formula $d=2t/\sqrt{gdl}$ , considering the categorization of very small effect size if *d* was lower than 0.20, small effect size if *d* varied from 0.20 to 0.49, medium effect size if *d* ranged from 0.50 to 0.79, and large effect size if *d* was equal or higher than 0.80 (Cohen, 1988; Calvo-Lobo et al., 2019; Marugán-Rubio et al., 2022).

Following the last aim of this research study, the influence of age and sex on the outcomes that showed effectiveness under this treatment in NSLBP patients was determined by analyses of covariance (ANCOVA). Multivariate linear regression analyses were performed to determine the influence and prediction of the statistically significant outcome measurement differences between both interventions according to the analyses described above (i.e., left diaphragm thickness at T^ins^ and T^ins-exp^, right and left PPTs) based on the intervention group, sex, and age. First, ANCOVAs for repeated measures with linear graphs were performed for each statistically significant outcome considering two groups (IMT and BFB + IMT groups) × two times (baseline and after intervention measurement moments) × covariables (age and sex) considering the *p*-value and F statistic according to the Greenhouse–Geisser correction if the Mauchly test rejected the sphericity completed with the partial eta squared coefficient (η_p_^2^), considering η_p_^2^ = 0.01 as a small effect size, η_p_^2^ = 0.06 as a medium effect size, and η_p_^2^ = 0.14 as a large effect size (Cohen, 1973; Blanca et al., 2017). Second, each linear regression analysis was performed for each statistically significant outcome using the “stepwise selection” method. Each regression coefficient (*R* ^2^) was calculated to determine the adjustment quality (Austin and Steyerberg, 2015). In addition, age (years), sex (female = 1; male = 2), and intervention group (IMT = 1; BFB + IMT = 2) were considered as independent variables for each linear regression analysis. Each outcome measurement that presented statistically significant differences between the intervention groups was included for each linear regression analysis as a dependent variable. The pre-set *F* probabilities were P_in_ and P_out_ of 0.05 and 0.10, respectively (Calvo-Lobo et al., 2019; Marugán-Rubio et al., 2022).

## 3 Results

### 3.1 Study sample and flow diagram

**FIGURE 3:**
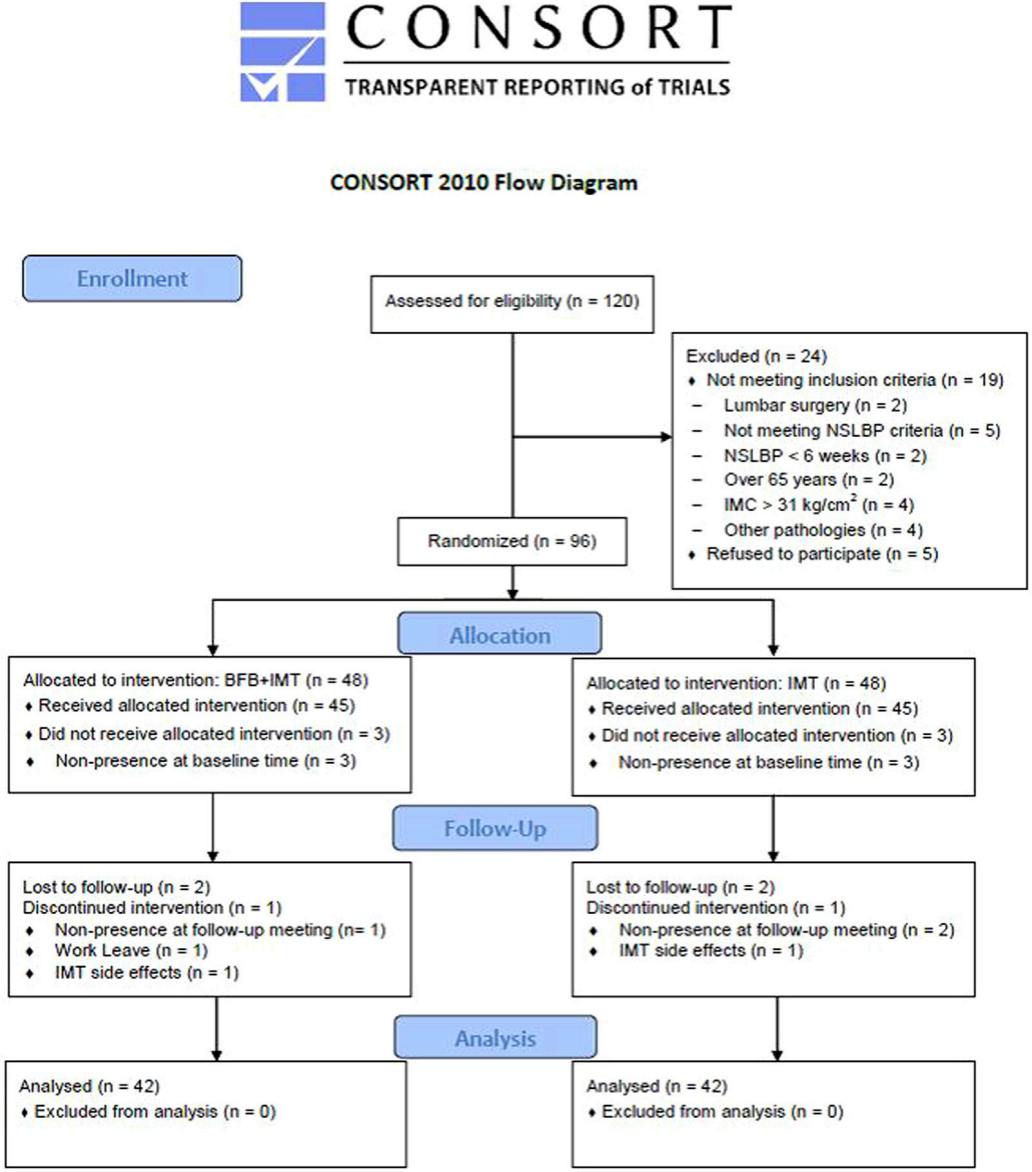
Flow diagram of the study course.

Of 120 patients with NSLBP assessed for eligibility, 24 participants were excluded secondary to lumbar surgery (*n* = 2), not meeting NSLBP criteria of the study (*n* = 5), NSLBP shorter than 6 weeks (*n* = 2), age over 65 years (*n* = 2), IMC greater than 31 kg/cm^2^ (*n* = 4), presence of other pathologies (n = 4), and refusal to participate (*n* = 5). Thus, a total of 96 patients with NSLBP were randomized into BFB + IMT (*n* = 48) and IMT (*n* = 48). In each group, three patients did not receive the allocated intervention due to not attending at the baseline time for the initial evaluation, and the remaining patients were assessed at baseline and received the allocated intervention in each group (*n* = 45). During the follow-up, three participants were lost in each group due to not attending follow-up meetings, work leave, and IMT side effects such as abdominal discomfort and dental problems during IMT. Finally, 84 patients with NSLBP were analyzed (*n* = 42 patients in each group), according to Figure 3.

### 3.2 Baseline descriptive data

The baseline sample was pair-matched by sex (*p* = 1.00) and comprised 90 patients with NSLBP divided into a BFB + IMT group (*n* = 45), which included 23 (51.11%) female and 22 (48.89%) male patients, and an IMT group (*n* = 45), which comprised 22 (48.89%) female and 23 (51.11%) male patients. The total sample showed a mean ± SD of age of 47.66 ± 11.10 years and normal BMI of 25.10 ± 3.16 kg/cm^2^, being homogeneous (*p* > 0.05) regarding the age, height, weight, BMI, and IPAQ scores between both groups. Furthermore, the IPAQ physical activity levels were similar (*p* = 0.391) between both groups because the BFB + IMT group included six sedentary patients, 14 participants with moderate activity, and 25 patients who performed vigorous physical activity, while the IMT group comprised six sedentary patents, 20 patients with moderate activity, and 19 patients who performed vigorous physical activity. Table 1 shows the baseline quantitative descriptive data of the BFB + IMT and IMT groups.

**TABLE 1:** Comparison of baseline descriptive data between BFB + IMT and IMT groups.

Baseline descriptive data (*n* = 90)	IMT (n = 45) mean ± SD (95% CI) [Range]	BFB + IMT (n = 45) Mean ± SD (95% CI) [Range]	*p*-value
Age (years)	49.50 ± 8.94 (46.71–52.28) [19.00–64.00]	45.83 ± 12.76 (41.85–48.81) [18.00–63.00]	0.495^†^
Height (cm)	170.07 ± 9.84 (167.00–173.14) [152.00–195.00]	170.86 ± 9.84 (167.00–173.14) [152.00–195.00]	0.642*
Weight (kg)	73.69 ± 13.90 (69.35–78.02) [49.00–100.00]	73.07 ± 13.30 (68.93–77.22) [49.00–105.00]	0.746*
BMI (kg/m^2^)	25.28 ± 3.01 (24.34–26.21) [19.38–30.67]	24.92 ± 3.33 (23.88–25.96) [16.76–30.46]	0.469*
IPAQ (METs/min/wk)	1917.95 ± 2414.19 (1421.71–3253.97) [160.00–6630.00]	2126.38 ± 1650.66 (1612.00–2640.77) [184.80–8586.00]	0.218^†^

*Abbreviations:* BFB, simultaneous bilateral visual biofeedback of the diaphragm by ultrasound imaging; CI, confidence interval; BMI, body mass index; IMT, inspiratory muscle training; IPAQ, International Physical Activity Questionnaire; METs/min/wk, metabolic equivalent index per minute per week. *p* < 0.05 was considered statistically significant for a 95% CI. * Student’s *t*-test was applied for independent samples. ^†^ Mann–Whitney *U* test was applied for independent samples.

### 3.3 Baseline respiratory outcomes

Although the total sample (*n* = 96) was randomized, only 90 patients were assessed at baseline due to the non-presence of six patients for the initial evaluation. This sample was homogenous for all respiratory outcomes at baseline because there were not statistically significant differences (*p* > 0.05) between both intervention groups for bilateral diaphragm thickness during normal breathing, respiratory muscle strength, and lung function spirometry parameters. These findings are presented in Table 2.

**TABLE 2:** Comparison of baseline respiratory outcomes between BFB + IMT and IMT groups.

Baseline respiratory outcomes (n = 90)	IMT (n = 45) mean ± SD (95% CI) [Range]	BFB + IMT (n = 45) Mean ± SD (95% CI) [Range]	*p*-value
Right diaphragm thickness at T^ins^ (cm)	0.21 ± 0.06 (0.19–0.22) [0.13–0.38]	0.20 ± 0.06 (0.19–0.22) [0.01–0.31]	0.601*
Right diaphragm thickness at T^exp^ (cm)	0.19 ± 0.05 (0.17–0.21) [0.12–0.35]	0.18 ± 0.04 (0.16–0.19) [0.11–0.30]	0.614^†^
Right diaphragm thickness at T^ins-exp^ (cm)	0.01 ± 0.03 (0.00–0.02) [−0.06–0.08]	0.02 ± 0.04 (0.01–0.04) [−0.17–0.11]	0.054*
Left diaphragm thickness at T^ins^ (cm)	0.22 ± 0.07 (0.20–0.24) [0.12–0.48]	0.21 ± 0.06 (0.19–0.23) [0.11–0.38]	0.455^†^
Left diaphragm thickness at T^exp^ (cm)	0.19 ± 0.05 (0.17–0.20) [0.08–0.33]	0.18 ± 0.05 (0.11–0.14) [0.16–0.20]	0.691*
Left diaphragm thickness at T^ins-exp^ (cm)	0.03 ± 0.04 (0.02–0.04) [−0.05–0.16]	0.02 ± 0.03 (0.01–0.03) [−0.04–0.18]	0.591^†^
MIP (cmH_2_O)	69.87 ± 31.76 (59.81–79.61) [17.67–153.33]	71.07 ± 28.96 (84.32–104.99) [62.05–80.10]	0.994^†^
MEP (cmH_2_O)	100.50 ± 39.40 (88.22–112.78) [27.33–174.67]	105.26 ± 41.99 (92.17–118.36) [37.67–185.67]	0.802^†^
FEV_1_ L)	2.98 ± 0.74 (2.75–3.22) [1.37–4.77]	2.99 ± 0.85 (2.91–3.54) [1.49–4.93]	0.907*
FVC L)	3.17 ± 0.83 (2.91–3.43) [1.39–5.46]	3.23 ± 1.01 (2.91–3.54) [1.49–6.03]	0.821*
FEV_1_/FVC (%)	94.06 ± 5.39 (92.38–95.74) [79.55–99.94]	93.70 ± 6.53 (91.67–95.74) [79.55–99.94]	0.955^†^

*Abbreviations:* BFB, simultaneous bilateral visual biofeedback of the diaphragm by ultrasound imaging; CI, confidence interval; FEV_1_, forced expiratory volume during 1 s; FVC, forced vital capacity; IMT, inspiratory muscle training; MEP, maximum expiratory pressure; MIP, maximum inspiratory pressure; T^ins^, maximum inspiration time; T^exp^, maximum expiration time. *p* < 0.05 was considered statistically significant for a 95% CI. * Student’s *t*-test was applied for independent samples. ^†^ Mann–Whitney *U* test was applied for independent samples.

### 3.4 Baseline clinical outcomes

The sample was also homogenous for all clinical outcomes at baseline because there were no significant differences (*p* > 0.05) between both groups for pain intensity, bilateral PPT of paraspinal muscles, disability, or quality of life for total scores and physical and mental health domains. These findings are presented in Table 3.

**TABLE 3:** Comparison of baseline clinical outcomes between BFB + IMT and IMT groups.

Baseline clinical outcome (n = 90)	IMT (n = 45) mean ± SD (95% CI) [Range]	BFB + IMT (n = 45) Mean ± SD (95% CI) [Range]	*p*-value
VAS (score)	4.59 ± 1.94 (3.98–5.19) [1.50–8.80]	5.10 ± 1.57 (4.61–5.59) [2.00–8.70]	0.544*
Right paraspinal muscles PPT (kg/cm^2^)	4.66 ± 2.06 (4.01–5.30) [1.40–10.00]	4.61 ± 1.59 (4.11–5.11) [1.57–8.33]	0.886*
Left paraspinal muscles PPT (kg/cm^2^)	4.64 ± 2.17 (3.96–5.32) [1.30–9.93]	4.54 ± 1.63 (4.03–5.05) [1.70–8.67]	0.932^†^
RMDQ (score)	3.78 ± 2.79 (2.91–4.65) [0.00–12.00]	3.69 ± 3.18 (3.69–5.68) [0.00–12.00]	0.297^†^
Physical health SF-12 (score)	15.83 ± 2.04 (15.19–16.47) [11.00–19.00]	15.26 ± 2.04 (14.62–15.90) [10.00–19.00]	0.424^†^
Mental health SF-12 (score)	20.14 ± 2.88 (19.24–21.04) [12.00–26.00]	20.09 ± 2.78 (19.22–20.96) [13.00–24.00]	0.987^†^
Total score SF-12 (score)	35.92 ± 4.22 (34.61–37.24) [23.00–44.00]	35.35 ± 3.98 (34.11–36.59) [23.00–41.00]	0.716^†^

*Abbreviations:* BFB, simultaneous bilateral visual biofeedback of the diaphragm by ultrasound imaging; CI, confidence interval; IMT, inspiratory muscle training; PPT, pressure pain threshold; RMDQ, Roland–Morris Disability Questionnaire; SF–12, 12-Item Short Form health questionnaire; VAS, visual analog scale. *p* < 0.05 was considered statistically significant for a 95% CI. * Student’s *t*-test was applied for independent samples. ^†^ Mann–Whitney *U* test was applied for independent samples.

### 3.5 Effectiveness of both interventions on primary outcomes

After 8 weeks of intervention, the BFB + IMT group showed statistically significant differences (*p* < 0.05) with an increase in the left hemidiaphragm thickness difference at T^ins^ with a medium effect size (*d* = 0.53) and T^ins-exp^ with a small effect size (*d* = 0.38) compared to the IMT group. The remaining diaphragm thickness differences after 8 weeks of both interventions did not show statistically significant differences (*p* > 0.05) with an effect size from very small to small (*d* = 0.14–0.38). These results are shown in Table 4.

**TABLE 4:** Effectiveness for primary outcome differences between BFB + IMT and IMT groups after 8 weeks.

Primary outcome difference after 8 weeks (n = 84)	IMT (n = 42) mean ± SD (95% CI) [Range]	BFB + IMT (n = 42) Mean ± SD (95% CI) [Range]	Cohen´s *d*	*p*-value
Right diaphragm thickness at T^ins^ (cm)	0.02 ± 0.07 (−0.003–0.04) [−0.15–0.20]	0.03 ± 0.07 (0.01–0.06) [−0.11–0.27]	0.14	0.318*
Right diaphragm thickness at T^exp^ (cm)	−0.02 ± 0.05 (−0.03–−0.003) [−0.16–0.12]	−0.001 ± 0.05 (−0.01–0.01) [−0.11–0.12]	0.38	0.104*
Right diaphragm thickness at T^ins-exp^ (cm)	0.04 ± 0.06 (0.02–0.06) [−0.07–0.20]	0.03 ± 0.06 (0.02–0.05) [−0.08–0.22]	0.16	0.747^†^
Left diaphragm thickness at T^ins^ (cm)	−0.01 ± 0.08 (−0.03–0.01) [−0.30–0.13]	0.03 ± 0.07 (0.01–0.05) [−0.08–0.17]	0.53	0.004*
Left diaphragm thickness at T^exp^ (cm)	−0.01 ± 0.05 (−0.03–0.003) [−0.21–0.09]	0.01 ± 0.06 (−0.01–0.03) [−0.16–0.16]	0.36	0.075*
Left diaphragm thickness at T^ins-exp^ (cm)	0.001 ± 0.05 (−0.01–0.01) [−0.16–0.09]	0.02 ± 0.05 (0.008–0.04) [−0.14–0.17]	0.38	0.045*

*Abbreviations:* BFB, simultaneous bilateral visual biofeedback of the diaphragm by ultrasound imaging; CI, confidence interval; IMT, inspiratory muscle training; T^ins^, maximum inspiration time; T^exp^, maximum expiration time. *p* < 0.05 was considered statistically significant for a 95% CI (**in bold**). * Student’s *t*-test was applied for independent samples. ^†^ Mann–Whitney *U* test was applied for independent samples.

### 3.6 Effectiveness of both interventions on other respiratory secondary outcomes

There were no statistically significant differences (*p* > 0.05) with an effect size from very small to small (*d* = 0.10–0.23) for the other respiratory secondary outcomes, including respiratory muscle strength and lung function spirometry parameters. These findings are shown in Table 5.

**TABLE 5:** Effectiveness for other respiratory secondary outcome differences between BFB + IMT and IMT groups after 8 weeks.

Respiratory secondary outcome difference after 8 weeks (n = 84)	IMT (n = 42) mean ± SD (95% CI) [Range]	BFB + IMT (n = 42) Mean ± SD (95% CI) [Range]	Cohen´s *d*	*p*-value
MIP (cmH_2_O)	26.39 ± 20.24 (20.09–32.70) [−4.33–69.66]	28.41 ± 18.40 (22.67–34.14) [2.67–74.00]	0.10	0.591^†^
MEP (cmH_2_O)	24.99 ± 25.82 (16.94–33.03) [−27.33–88.00]	30.38 ± 43.04 (16.96–43.79) [−18.33–88.00]	0.15	0.623^†^
FEV_1_ (L)	0.17 ± 0.48 (0.14–0.46) [−0.66–2.07]	0.12 ± 0.34 (0.01–0.23) [−0.46–0.83]	0.12	0.522^†^
FVC (L)	0.36 ± 0.63 (0.17–0.56) [−0.68–2.48]	0.23 ± 0.47 (0.08–0.38) [−0.68–1.49]	0.23	0.816^†^
FEV_1_/FVC (%)	−3.59 ± 6.61 (−5.65–−1.53) [−23.64–5.18]	−2.88 ± 6.94 (−5.04–−0.71) [−27.62–8.56]	0.10	0.681^†^

*Abbreviations:* BFB, simultaneous bilateral visual biofeedback of the diaphragm by ultrasound imaging; CI, confidence interval; FEV_1_, forced expiratory volume during 1 s; FVC, forced vital capacity; IMT, inspiratory muscle training; MEP, maximum expiratory pressure; MIP, maximum inspiratory pressure. *p* < 0.05 was considered statistically significant for a 95% CI. ^†^ Mann–Whitney *U* test was applied for independent samples.

### 3.7 Effectiveness of both interventions on clinical secondary outcomes

After 8 weeks of intervention, the BFB + IMT group presented statistically significant differences (*p* < 0.01) with an increased right and left PPT with a medium effect size (*d* = 0.71–0.74) with respect to the IMT group. The remaining clinical outcome differences did not show statistically significant differences (*p* > 0.05), with an effect size from very small to small (*d* = 0.02–0.46). These findings are presented in Table 6.

**TABLE 6:** Effectiveness for clinical secondary outcome differences between BFB + IMT and IMT groups after 8 weeks.

Clinical secondary outcome difference after 8 weeks (n = 84)	IMT (n = 42) mean ± SD (95% CI) [Range]	BFB + IMT (n = 42) Mean ± SD (95% CI) [Range]	Cohen´s *d*	*p*-value
VAS (score)	−2.58 ± 2.24 (−3.28–−1.88) [−8.80–3.30]	−2.48 ± 2.23 (−3.17–1.78) [−6.40–2.40]	0.04	0.842*
Right paraspinal muscles PPT (kg/cm^2^)	1.09 ± 1.48 (0.63–1.55) [−1.77–6.03]	2.16 ± 1.40 (1.72–2.60) [−1.46–5.73]	0.74	0.001*
Left paraspinal muscles PPT (kg/cm^2^)	1.16 ± 1.51 (0.69–1.63) [−1.77–6.50]	2.27 ± 1.59 (1.78–2.77) [−2.07–5.73]	0.71	0.002*
RMDQ (score)	−2.00 ± 2.10 (−2.65–−1.34) [−8.00–2.00]	−1.83 ± 3.44 (−2.60–−0.76) [−11.00–9.00]	0.05	0.846^†^
Physical health SF-12 (score)	1.37 ± 1.69 (0.82–1.88) [−2.00–5.00]	1.42 ± 2.27 (0.71–2.13) [−4.00–7.00]	0.02	0.809^†^
Mental health SF-12 (score)	2.21 ± 2.59 (1.40–3.02) [-3.00–10.00]	1.07 ± 2.36 (0.33–1.80) [-3.00–6.00]	0.46	0.051^†^
Total score SF-12 (score)	3.61 ± 3.21 (2.61–4.62) [−2.00–11.00]	2.50 ± 3.96 (1.26–3.73) [−6.00–10.00]	0.30	0.124^†^

*Abbreviations:* BFB, simultaneous bilateral visual biofeedback of the diaphragm by ultrasound imaging; CI, confidence interval; IMT, inspiratory muscle training; PPT, pressure pain threshold; RMDQ, Roland–Morris Disability Questionnaire; SF-12, 12-Item Short Form health questionnaire; VAS, visual analog scale. *p* < 0.05 was considered statistically significant for a 95% CI (**in bold**). * Student’s *t*-test was applied for independent samples. ^†^ Mann–Whitney *U* test was applied for independent samples.

### 3.8 Influence of age and sex in the effectiveness of intervention

The age and sex influence on the outcome differences (i.e., left diaphragm thickness at T^ins^ and T^ins-exp^, right and left PPTs) that showed statistically significant differences after 8 weeks of BFB + IMT *versus* IMT in NSLBP patients was analyzed by ANCOVAs for repeated-measures according to the Greenhouse–Geisser correction and predicted by multivariate linear regression analyses based on age, sex, and group as independent variables.

**FIGURE 4:**
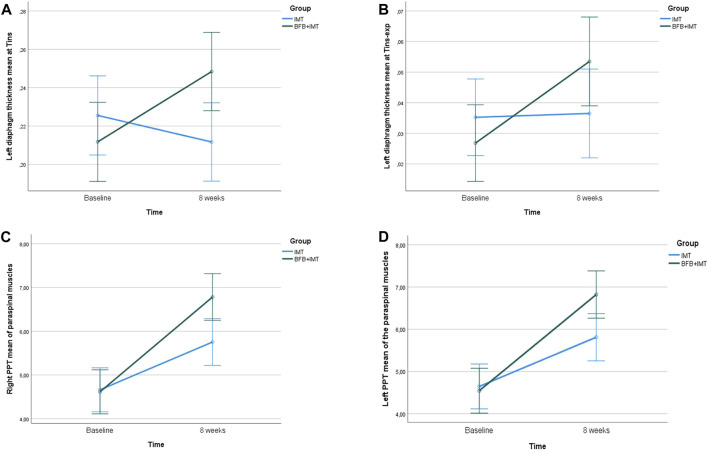
Linear graphs for the **(A)** left hemidiaphragm thickness at T^ins^, **(B)** left hemidiaphragm thickness at T^ins-exp^, **(C)** right PPT of the paraspinal muscles, and **(D)** left PPT of the paraspinal muscles after 8 weeks of BFB + IMT *versus* IMT and covariate age and sex. The covariates in the model were evaluated at the following values: age = 47.66; sex = 1.50. Error bars: 95% CI. *Abbreviations*: BFB, simultaneous bilateral visual biofeedback of the diaphragm by ultrasound imaging; CI, confidence interval; IMT, inspiratory muscle training; PPT, pressure pain threshold; T^ins^, maximum inspiration time; T^exp^, maximum expiration time.

First, the interactions of time × group (*p* = 0.003; F_(1,81)_ = 9.739; η_p_^2^ = 0.107) and time × sex (*p* = 0.007; F_(1,81)_ = 7.756; η_p_^2^ = 0.087) were statistically significant with medium effect sizes for the left hemidiaphragm thickness difference at T^ins^ (Figure 4A). However, the interaction of age × group (*p* = 0.233; F_(1,81)_ = 1.442; η_p_^2^ = 0.018) was not statistically significant with a small effect size. A linear regression model (*R* ^2^ = 0.178) predicted a higher left hemidiaphragm thickness difference at T^ins^ based on the BFB + IMT group (*R* ^2^ = 0.099; β = 0.050; F_(1,82)_ = 8.997; *p* = 0.004) and male sex (*R* ^2^ = 0.079; β = 0.045; F_(1,81)_ = 7.756; *p* = 0.007).

Second, the interaction of time × group (*p* = 0.045; F_(1,81)_ = 4.130; η_p_^2^ = 0.049) was also statistically significant with a small effect size, but neither time × sex (*p* = 0.447; F_(1,81)_ = 0.585; η_p_^2^ = 0.007) or time × age (*p* = 0.084; F_(1,81)_ = 3.052; η_p_^2^ = 0.037) with small effect sizes was statistically significant for the left hemidiaphragm thickness difference at T^ins-exp^ (Figure 4B). A linear regression model (*R* ^2^ = 0.052) predicted a greater left hemidiaphragm thickness difference at T^ins-exp^ based on younger age (*R* ^2^ = 0.052; β = −0.001; F_(1,82)_ = 4.540; *p* = 0.036).

Third, time × group interaction (*p* = 0.001; F_(1,81)_ = 11.501; η_p_^2^ = 0.124) was also significant with a medium effect size, although neither time × sex interaction (*p* = 0.799; F_(1,81)_ = 0.065; η_p_^2^ = 0.001) nor time × age interaction (*p* = 0.755; F_(1,81)_ = 0.098; η_p_^2^ = 0.001) with small effect sizes, was effective for the right PPT of the paraspinal muscles (Figure 4C). The multivariate analysis did not display a valid linear regression model due to a non-significant constant *p*-value according to pre-set *F* probability considering P_in_ and P_out_ of 0.05 and 0.10, respectively.

Lastly, the interaction of time × group (*p* = 0.002; F_(1,81)_ = 10.587; η_p_^2^ = 0.116) also presented significant differences with a medium effect size, but neither time × sex interaction (*p* = 0.952; F_(1,81)_ = 0.004; η_p_^2^ = 0.000) nor time × age interaction (*p* = 0.292; F_(1,81)_ = 1.124; η_p_^2^ = 0.014) with small effect sizes, were effective for the left PPT of the paraspinal muscles (Figure 4D). In line with the last analysis, the multivariate analysis did not display any valid linear regression model following the non-significant constant *p*-value of the pre-established *F* probability values for P_in_ of 0.05 and P_out_ of 0.10.

## 4 Discussion

Here, our research group presented the first randomized clinical trial to determine the effectiveness of simultaneous bilateral visual biofeedback of the diaphragm muscle by ultrasonography through a reliable and novel proposed thoracic orthosis that allowed the simultaneous reeducation of both the right and left hemidiaphragms during normal breathing in conjunction with high-intensity IMT in patients with NSLBP (Molina-Hernández et al., 2023).

According to the primary outcomes, the addition of simultaneous bilateral visual biofeedback about the diaphragm muscle by ultrasonography to IMT increased the left hemidiaphragm thickness at maximum inspiration and during normal breathing with respect to maximum expiration. However, the right hemidiaphragm thickness did not reach significant differences during normal breathing. This outcome may be because the sample size of our study was based on the left hemidiaphragm thickness during maximum inspiration, and this hemidiaphragm was claimed to play a key role in postural function (Celli, 1989; Hruska, 1997; Terada et al., 2016). Previously, the unilateral visual biofeedback from each hemidiaphragm separately did not produce any increase in the diaphragm thickness during normal breathing (Marugán-Rubio et al., 2022). Secondary to these findings, our research group registered a novel thoracic device to permit the bilateral fixation of two ultrasound proves to allow the simultaneous bilateral reeducation of the diaphragm muscle (Molina-Hernández et al., 2023), which reinforced the understanding that the inspiratory muscle activity of both the right and left hemidiaphragms seemed to be bilaterally and simultaneously performed (Boussuges et al., 2009), avoiding the distortion according to non-normal changes in diaphragm geometry (Bellemare et al., 1986).

Regarding the other respiratory outcomes, the proposed interventions did not present significant differences in respiratory muscle strength by MIP and MEP or lung function by spirometry parameters. Nevertheless, the unilateral visual biofeedback of each hemidiaphragm separately improved lung function in addition to IMT, which was predicted by a FEV_1_ increase in athletes with NSLBP (Marugán-Rubio et al., 2022). These differences could be secondary to the unilateral distortion of the diaphragm geometry (Bellemare et al., 1986), and the isolated reeducation of each hemidiaphragm separately could have better effects on chest wall expansion, which was previously correlated with FEV_1_ increase (Calvo-Lobo et al., 2018).

Considering the secondary clinical outcomes, the use of simultaneous bilateral biofeedback of the diaphragm bilaterally increased the PPT of the paraspinal muscles, reducing muscle mechanosensitivity in conjunction with IMT in patients with NSLBP, although these improvements were not presented after unilateral and separate visual reeducation of right and left hemidiaphragms (Marugán-Rubio et al., 2022). This fact may be secondary to the simultaneous core muscle co-activation, which may be improved after the reeducation of both hemidiaphragms at the same time during normal breathing (Hodges et al., 1997). The other clinical outcomes, such as pain intensity, disability, and quality of life, did not present significant differences after simultaneous bilateral biofeedback in line with the unilateral reeducation of the diaphragm muscle in addition to IMT (Marugán-Rubio et al., 2022). Some possible reasons that explain these findings may be that the high-intensity IMT presented notable clinical improvements in an isolated manner, and low-intensity or sham IMT could have shown better effects of the visual biofeedback more clearly (Janssens et al., 2015). In addition, the self-reported clinical outcome differences that remained unaffected by the intervention could have been influenced by errors of measurement according to the SEM and MDC values of pain (Alghadir et al., 2018), disability (Jenks et al., 2022), and quality of life (Clement et al., 2019). Thus, future studies should be controlled, including a sham IMT intervention, to provide clear clinical differences and deepen knowledge of the effectiveness of the simultaneous bilateral visual biofeedback of the diaphragm (Janssens et al., 2015).

Lastly, the sex-based and aged-based analyses showed that these covariables influenced our findings regarding the left diaphragm thickness increase at T^ins^ and T^ins-exp^ during normal breathing, respectively, after 8 weeks of bilateral visual biofeedback of the diaphragm muscle in conjunction with high-intensity IMT. Indeed, a higher increase of the left diaphragm muscle at T^ins^ was predicted by the BFB + IMT group and male sex. In addition, a higher increase of the left diaphragm muscle at T^ins-exp^ was predicted by a younger age. These findings reinforced the fact that sex-based and aged-based fatigability of the diaphragm muscle may influence exercise performance (Fogarty et al., 2019; Andrew Harry Ramsook BPHE, 2021).

### 4.1 Further studies

Further studies should control the effectiveness of the proposed simultaneous bilateral reeducation of the diaphragm, including a third arm with a sham IMT intervention (Janssens et al., 2015). In addition, other musculoskeletal conditions could benefit from the simultaneous reeducation of both hemidiaphragms. For example, women with fibromyalgia showed positive effects in respiratory efficiency and quality of life after IMT, and the proposed simultaneous bilateral diaphragmatic reeducation could improve these results (Tomas-Carus et al., 2022).

### 4.2 Limitations

The lack of a control group with a sham IMT may be considered the main limitation of our study (Vicente-Campos et al., 2021). High-intensity IMT was proposed in both groups because this intervention was shown to be more effective than low-intensity IMT in patients with NSLBP, and other IMT intensities should be considered in future studies (Janssens et al., 2015). The authors acknowledge that the specificity of the inclusion and exclusion criteria, which could potentially restrict the generalizability of the study’s findings, could be a limitation. Specifically, criteria such as an age limitation of 65 years and older and a BMI greater than 31 kg/cm^2^ may be noteworthy because it is not uncommon for older and obese patients to experience NSLBP (Maher et al., 2017).

## 5 Conclusion

In conclusion, the simultaneous bilateral visual diaphragm biofeedback intervention by ultrasonography added to IMT increased the left diaphragmatic thickness during inspiration *versus* the isolated application of IMT in patients with NSLBP. Furthermore, this simultaneous bilateral visual diaphragm biofeedback intervention plus IMT also increased the bilateral PPT of the paraspinal muscles with respect to isolated IMT in patients with NSLBP. Lastly, the left diaphragmatic thickness increase during inspiration after 8 weeks was positively influenced and predicted by the addition of simultaneous bilateral visual diaphragm biofeedback to IMT, male sex, and younger age in NSLBP patients. Overall, the proposed intervention demonstrated novelty, particularly in its exploration of simultaneous bilateral re-education of the diaphragmatic muscle supporting clinical implications in patients with NSLBP, and future research studies should be carried out in other musculoskeletal conditions.

## Data Availability

The original contributions presented in the study are included in the article/Supplementary Material; further inquiries can be directed to the corresponding author.
