# Supplementary material for: Effectiveness of simultaneous bilateral visual diaphragm biofeedback under low back pain: influence of age and sex
Source: Front Physiol. 2024 Jul 9;15:1407594. doi: 10.3389/fphys.2024.1407594 (PMC11263192; doi:10.3389/fphys.2024.1407594)
Supplement: Supplementary file 1 [file DataSheet1.PDF]

Participant	Group	Gender	Age	Weigth	Heigth	BMI	VAS_PRE	MIP_PRE	MEP_PRE
1,00	2,00	2,00	24,00	105,00	1,93	28,19	5,70	84,33	106,33
2,00	2,00	2,00	59,00	90,00	1,83	26,87	5,00	58,67	68,33
3,00	2,00	2,00	54,00	90,00	1,78	28,41	5,20	43,00	65,33
4,00	1,00	2,00	52,00	90,00	1,82	27,17	4,00	48,00	83,67
5,00	1,00	1,00	56,00	60,00	1,59	23,73	2,50	38,67	56,00
6,00	1,00	2,00	56,00	70,00	1,80	21,60	8,80	71,33	112,33
7,00	1,00	2,00	19,00	100,00	1,95	26,30	3,70	87,33	112,33
8,00	1,00	2,00	18,00	60,00	1,62	22,86	5,70	51,67	60,33
9,00	2,00	2,00	18,00	70,00	1,70	24,22	6,00	40,33	76,00
10,00	2,00	2,00	41,00	79,00	1,80	24,38	4,40	80,00	145,33
11,00	2,00	1,00	24,00	62,00	1,71	21,20	4,60	59,00	84,33
12,00	2,00	1,00	59,00	73,00	1,63	27,48	2,80	62,33	70,33
13,00	1,00	1,00	54,00	78,00	1,72	26,37	2,80	45,33	103,00
14,00	2,00	2,00	24,00	80,00	1,85	23,37	7,00	64,33	153,33
15,00	1,00	2,00	40,00	100,00	1,83	29,86	3,10	86,00	130,67
16,00	1,00	1,00	56,00	78,00	1,66	28,31	6,80	31,33	51,33
17,00	2,00	2,00	28,00	70,00	1,85	20,45	2,80	85,67	112,00
18,00	1,00	1,00	48,00	63,00	1,60	24,61	4,00	62,67	64,00
19,00	1,00	2,00	45,00	80,00	1,80	24,69	7,50	76,33	117,67
20,00	1,00	1,00	53,00	73,00	1,65	26,81	6,50	88,67	96,67
21,00	2,00	2,00	55,00	100,00	1,84	29,54	5,40	102,00	121,33
22,00	2,00	1,00	58,00	71,20	1,63	26,80	5,40	80,33	150,33
23,00	1,00	1,00	58,00	60,00	1,62	22,86	7,20	60,33	79,00
24,00	1,00	1,00	37,00	50,00	1,60	19,53	1,50	40,33	51,00
25,00	1,00	1,00	46,00	51,00	1,61	19,68	6,00	43,00	74,33
26,00	1,00	1,00	46,00	78,00	1,68	27,64	3,90	60,00	83,33
27,00	1,00	1,00	47,00	80,00	1,62	30,48	2,60	86,67	76,33
28,00	2,00	2,00	42,00	82,00	1,95	21,56	4,80	82,00	154,33
29,00	2,00	1,00	40,00	67,00	1,72	22,65	4,70	29,67	40,00
30,00	2,00	1,00	48,00	50,00	1,53	21,36	2,00	69,67	114,67
31,00	2,00	1,00	51,00	55,00	1,68	19,49	4,80	62,33	78,67
32,00	1,00	1,00	49,00	65,00	1,62	24,77	8,90	48,67	87,33
33,00	1,00	1,00	44,00	70,00	1,59	27,69	5,30	61,33	60,00
34,00	2,00	2,00	42,00	72,00	1,72	24,34	5,60	118,67	185,67
35,00	2,00	1,00	39,00	60,00	1,65	22,04	5,10	41,67	60,67
36,00	1,00	1,00	44,00	72,00	1,68	25,51	4,00	59,67	123,33
37,00	1,00	2,00	52,00	97,00	1,78	30,61	6,20	108,67	175,67
38,00	2,00	1,00	57,00	58,00	1,69	20,31	3,00	33,00	78,00
39,00	2,00	1,00	46,00	75,00	1,63	28,23	6,30	32,67	46,33
40,00	1,00	1,00	50,00	49,00	1,59	19,38	1,60	40,00	105,00
41,00	1,00	1,00	55,00	53,00	1,63	19,95	5,30	62,33	77,33
42,00	1,00	1,00	49,00	56,00	1,62	21,34	4,50	52,67	71,00
43,00	1,00	1,00	44,00	65,00	1,65	23,88	6,20	17,67	27,33
44,00	1,00	2,00	51,00	82,00	1,78	25,88	3,20	55,67	52,67
45,00	2,00	2,00	47,00	82,00	1,83	24,49	6,00	107,00	162,00
46,00	2,00	2,00	50,00	81,00	1,72	27,38	2,30	147,67	171,00
47,00	1,00	2,00	54,00	70,00	1,75	22,86	6,70	43,00	111,00
48,00	1,00	1,00	52,00	62,00	1,55	25,81	8,20	37,00	56,67
49,00	2,00	2,00	51,00	83,00	1,72	28,06	4,90	98,67	128,33

50,00	2,00	2,00	51,00	82,00	1,77	26,17	6,60	98,33	181,33
51,00	2,00	1,00	50,00	62,00	1,57	25,15	4,10	91,00	103,33
52,00	2,00	1,00	45,00	55,00	1,58	22,03	8,70	46,67	76,67
53,00	2,00	1,00	56,00	80,00	1,65	29,38	3,30	67,00	86,00
54,00	1,00	2,00	64,00	90,00	1,73	30,07	4,10	104,33	167,00
55,00	2,00	1,00	62,00	68,00	1,65	24,98	5,00	35,33	75,67
56,00	2,00	1,00	62,00	61,00	1,62	23,24	6,30	35,00	78,67
57,00	1,00	1,00	51,00	73,00	1,65	26,81	6,30	100,33	112,33
58,00	1,00	2,00	47,00	86,00	1,78	27,14	2,90	112,33	152,33
59,00	2,00	1,00	54,00	54,00	1,62	20,58	3,00	59,33	81,00
60,00	2,00	2,00	57,00	75,00	1,73	25,06	3,00	71,67	151,33
61,00	1,00	1,00	60,00	71,00	1,66	25,77	1,80	36,33	68,00
62,00	1,00	2,00	40,00	74,00	1,71	25,31	3,80	108,00	152,33
63,00	1,00	1,00	29,00	51,00	1,53	21,79	3,90	48,00	58,33
64,00	2,00	1,00	62,00	56,00	1,61	21,60	3,70	35,67	45,00
65,00	2,00	1,00	54,00	77,00	1,59	30,46	5,10	92,67	114,33
66,00	1,00	2,00	54,00	75,00	1,70	25,95	5,10	33,67	62,33
67,00	1,00	2,00	55,00	89,00	1,79	27,78	5,50	55,00	115,33
68,00	1,00	2,00	59,00	80,00	1,70	27,68	5,70	71,00	129,00
69,00	1,00	2,00	45,00	71,00	1,78	22,41	4,00	86,00	125,33
70,00	2,00	1,00	55,00	74,00	1,68	26,22	7,20	33,67	45,00
71,00	2,00	2,00	45,00	73,00	1,80	22,53	0,70	72,00	110,00
72,00	2,00	1,00	27,00	76,00	1,63	28,60	3,10	74,00	83,33
73,00	2,00	2,00	63,00	75,00	1,68	26,57	7,40	41,00	82,67
74,00	1,00	2,00	42,00	88,00	1,84	25,99	1,50	133,00	158,33
75,00	2,00	1,00	38,00	54,00	1,63	20,32	7,50	44,67	37,67
76,00	2,00	2,00	55,00	73,00	1,74	24,11	6,90	71,67	161,33
77,00	2,00	1,00	55,00	70,00	1,65	25,71	4,50	55,00	83,00
78,00	2,00	1,00	42,00	76,00	1,58	30,44	6,10	58,00	100,67
79,00	1,00	1,00	54,00	54,00	1,52	23,37	6,90	37,00	64,33
80,00	2,00	1,00	46,00	63,00	1,67	22,59	8,20	47,33	80,33
81,00	2,00	2,00	55,00	94,00	1,82	28,38	4,30	84,67	108,67
82,00	2,00	2,00	24,00	49,00	1,71	16,76	5,00	64,33	69,33
83,00	2,00	2,00	27,00	90,00	1,73	30,07	4,90	118,33	162,67
84,00	2,00	2,00	31,00	83,00	1,78	26,20	6,00	142,67	164,67
85,00	1,00	2,00	64,00	95,00	1,85	27,76	2,30	102,33	168,33
86,00	1,00	2,00	44,00	78,00	1,69	27,31	3,50	153,33	162,33
87,00	1,00	2,00	41,00	73,00	1,82	22,04	7,00	137,33	174,67
88,00	1,00	2,00	54,00	86,00	1,77	27,45	3,90	57,33	94,67
89,00	1,00	2,00	64,00	76,00	1,76	24,54	2,40	85,00	120,67
90,00	1,00	2,00	56,00	95,00	1,76	30,67	6,40	112,33	159,67

FVC_PRE	FEV1_PRE	FEV1_FVC_	PPT_Right_PRE	PPT_Left_PRE	IPAQ_Category	IPAQ
3,95	3,94	99,85	5,27	4,50	3,00	4572,00
4,46	3,86	86,68	7,40	8,13	3,00	3342,00
4,43	3,73	84,10	2,73	2,60	2,00	1080,00
4,20	3,76	89,54	4,57	4,80	2,00	1386,00
2,33	2,32	99,47	4,80	4,87	2,00	1554,00
4,16	4,11	98,82	5,73	6,10	1,00	396,00
4,80	4,77	99,30	5,33	5,70	3,00	6171,00
2,32	2,31	99,57	3,97	4,43	2,00	1245,00
2,74	2,74	99,94	5,77	5,93	3,00	8586,00
6,03	4,93	81,67	3,93	4,07	3,00	1500,00
3,50	3,46	98,90	4,27	4,27	3,00	2133,00
2,34	2,27	97,19	1,57	1,70	3,00	2514,00
2,30	2,29	99,41	6,33	6,80	2,00	1116,00
3,90	3,90	99,94	2,67	2,83	3,00	2719,00
2,98	2,95	99,25	4,23	4,20	3,00	1519,50
2,43	2,25	92,57	2,27	2,33	2,00	1386,00
2,20	2,20	99,79	5,40	5,67	3,00	2106,00
3,34	3,18	95,25	3,80	3,20	1,00	240,00
3,50	2,65	75,80	4,30	3,63	2,00	1462,00
2,78	2,63	94,70	5,00	4,70	2,00	876,00
3,61	3,12	86,37	4,37	4,50	1,00	405,00
2,61	2,27	87,13	3,70	4,07	3,00	1626,00
2,51	2,42	96,31	2,40	3,00	2,00	1173,00
3,09	2,85	92,21	5,13	5,23	2,00	1404,00
1,39	1,37	99,00	1,93	2,57	3,00	2559,00
3,09	3,06	99,13	1,90	1,97	3,00	1706,00
3,17	2,78	87,44	4,20	4,03	1,00	160,00
4,46	3,65	82,56	8,33	8,67	1,00	184,80
2,51	2,51	99,83	2,83	3,67	2,00	1404,00
1,49	1,49	99,45	6,00	6,40	3,00	3066,00
2,73	2,68	98,19	5,23	3,47	3,00	1685,00
2,99	2,93	98,29	2,83	3,33	2,00	693,00
2,08	2,03	98,04	4,13	3,93	2,00	924,00
4,47	4,14	92,70	5,17	5,00	2,00	1413,00
3,02	2,80	92,79	5,73	6,33	3,00	2106,00
3,86	3,69	95,53	3,70	3,00	1,00	480,00
3,80	3,68	97,02	7,07	6,77	3,00	1584,00
2,89	2,28	78,90	4,20	3,93	3,00	1905,00
2,81	2,39	85,13	1,67	2,13	3,00	4950,00
3,96	3,60	92,33	4,10	4,30	3,00	2346,00
2,37	2,25	94,91	2,80	2,60	3,00	2592,00
1,90	1,88	99,04	2,10	2,73	3,00	2586,00
3,26	3,12	95,54	1,40	1,30	2,00	924,00
3,54	3,54	99,94	5,90	5,90	2,00	678,00
4,09	4,05	99,01	6,03	5,13	2,00	1080,00
3,92	3,91	99,93	5,93	5,47	3,00	2346,00
1,99	1,99	99,92	1,93	1,50	3,00	5706,00
2,57	2,34	91,20	3,63	3,03	2,00	1386,00
3,64	3,63	99,75	6,93	6,87	3,00	4092,00

3,95	3,82	96,90	5,67	5,33	2,00	990,00
2,72	2,68	98,87	4,67	4,53	2,00	1386,00
3,32	2,99	90,08	1,60	1,90	3,00	5544,00
2,05	2,04	99,42	2,80	2,63	3,00	2772,00
2,49	2,31	92,71	7,47	8,73	1,00	396,00
2,01	1,72	85,89	2,70	2,80	3,00	2646,00
3,19	2,53	79,55	3,73	4,20	2,00	892,50
3,65	3,52	87,58	5,43	4,53	3,00	1830,00
1,87	1,87	99,76	6,40	6,20	3,00	6630,00
1,69	1,68	99,43	5,57	3,50	1,00	480,00
4,81	3,94	81,87	6,83	6,83	2,00	1440,00
3,50	3,19	90,51	4,37	3,90	2,00	693,00
5,46	4,60	84,03	4,10	3,93	2,00	1116,00
2,70	2,64	83,78	4,60	4,50	2,00	693,00
1,70	1,69	99,35	2,90	3,37	1,00	480,00
2,49	2,41	97,01	2,97	3,23	2,00	1440,00
3,65	3,50	96,14	2,80	2,67	2,00	1386,00
4,00	3,63	90,84	8,87	9,00	3,00	1626,00
3,84	3,57	93,02	2,90	3,10	3,00	2079,00
3,06	3,05	99,70	6,47	5,80	3,00	3336,00
1,93	1,84	95,47	5,10	5,27	2,00	1272,00
3,10	3,08	99,50	7,43	6,80	2,00	1386,00
2,50	2,48	98,86	4,43	4,17	1,00	198,00
2,80	2,62	93,33	5,07	5,30	2,00	693,00
3,28	3,23	98,55	5,83	6,87	2,00	1039,50
1,61	1,57	97,63	4,10	3,80	3,00	3252,00
4,53	3,83	84,48	7,13	7,10	3,00	2632,00
3,37	3,07	91,08	4,90	4,70	3,00	1866,00
3,12	2,72	86,95	2,60	2,63	1,00	396,00
2,86	2,54	88,88	2,00	2,03	1,00	594,00
3,15	2,79	88,49	5,50	6,17	2,00	942,00
3,51	3,15	89,97	4,17	4,07	2,00	693,00
3,10	3,09	99,53	2,53	2,03	3,00	4638,00
4,23	4,09	96,53	4,83	4,07	3,00	3852,00
3,82	3,80	99,26	4,73	3,97	3,00	2844,00
3,11	3,00	96,58	3,87	3,50	3,00	4464,00
3,19	2,96	93,03	6,87	7,40	3,00	4452,00
4,13	4,09	99,01	6,13	5,73	3,00	3465,00
3,50	3,24	92,65	6,70	5,97	3,00	3465,00
3,82	3,33	87,22	10,00	9,90	3,00	1866,00
3,73	3,44	92,11	9,33	9,93	2,00	693,00

SF12_Physical_DIRECT_SCORES_PRE	SF12_Mentall_DIRECT_SCORES_PRE
14,00	24,00
16,00	21,00
16,00	19,00
17,00	20,00
16,00	19,00
16,00	21,00
18,00	25,00
14,00	19,00
18,00	21,00
15,00	24,00
16,00	20,00
16,00	22,00
15,00	18,00
16,00	23,00
16,00	17,00
13,00	17,00
18,00	23,00
18,00	20,00
19,00	22,00
12,00	19,00
14,00	21,00
14,00	19,00
18,00	26,00
18,00	22,00
14,00	21,00
14,00	21,00
14,00	17,00
17,00	20,00
13,00	18,00
17,00	21,00
17,00	24,00
6,00	12,00
15,00	21,00
17,00	18,00
15,00	18,00
18,00	21,00
12,00	16,00
10,00	16,00
18,00	17,00
17,00	23,00
13,00	20,00
18,00	25,00
14,00	20,00
16,00	22,00
14,00	20,00
15,00	20,00
13,00	23,00
14,00	17,00
19,00	22,00

14,00	20,00
15,00	22,00
14,00	19,00
17,00	24,00
16,00	21,00
15,00	21,00
15,00	18,00
12,00	18,00
16,00	20,00
16,00	21,00
18,00	23,00
15,00	14,00
17,00	20,00
17,00	23,00
15,00	16,00
12,00	20,00
17,00	19,00
14,00	19,00
17,00	21,00
16,00	16,00
15,00	24,00
16,00	18,00
15,00	15,00
11,00	16,00
19,00	22,00
10,00	13,00
13,00	18,00
17,00	23,00
13,00	20,00
11,00	12,00
17,00	20,00
11,00	23,00
17,00	14,00
16,00	17,00
18,00	19,00
15,00	23,00
18,00	16,00
18,00	20,00
18,00	21,00
17,00	20,00
16,00	24,00

SF12_Total_DIRECT_SCORES_PRE	Rolland_Morris_PRE	Tins_Right_PRE	Texp_Right_PRE
38,00	11,00	0,21	0,19
37,00	3,00	0,20	0,16
35,00	0,00	0,30	0,27
37,00	2,00	0,13	0,15
35,00	7,00	0,13	0,15
37,00	0,00	0,23	0,25
43,00	2,00	0,38	0,31
33,00	6,00	0,14	0,10
39,00	3,00	0,22	0,16
39,00	4,00	0,28	0,19
36,00	3,00	0,21	0,18
38,00	5,00	0,00	0,17
33,00	12,00	0,22	0,18
39,00	2,00	0,12	0,11
33,00	4,00	0,24	0,19
30,00	10,00	0,17	0,16
41,00	3,00	0,19	0,20
38,00	2,00	0,33	0,25
39,00	2,00	0,19	0,17
31,00	10,00	0,30	0,23
35,00	11,00	0,28	0,21
33,00	2,00	0,17	0,12
44,00	5,00	0,23	0,28
40,00	5,00	0,18	0,15
35,00	4,00	0,19	0,19
35,00	3,00	0,19	0,23
31,00	4,00	0,17	0,15
37,00	5,00	0,25	0,18
31,00	5,00	0,22	0,22
38,00	1,00	0,24	0,16
41,00	3,00	0,23	0,19
18,00	12,00	0,29	0,29
36,00	2,00	0,29	0,35
35,00	6,00	0,17	0,16
33,00	3,00	0,30	0,20
39,00	9,00	0,27	0,22
28,00	5,00	0,28	0,27
26,00	4,00	0,35	0,20
35,00	2,00	0,36	0,18
40,00	3,00	0,16	0,20
33,00	2,00	0,13	0,12
43,00	2,00	0,19	0,16
34,00	2,00	0,20	0,16
38,00	2,00	0,21	0,22
34,00	11,00	0,26	0,20
35,00	3,00	0,31	0,30
36,00	5,00	0,17	0,15
31,00	4,00	0,16	0,12
41,00	1,00	0,24	0,20

34,00	3,00	0,25	0,23
37,00	6,00	0,29	0,20
33,00	6,00	0,26	0,26
41,00	5,00	0,18	0,19
37,00	4,00	0,15	0,14
36,00	4,00	0,17	0,16
33,00	12,00	0,20	0,15
30,00	4,00	0,20	0,14
36,00	1,00	0,20	0,16
37,00	3,00	0,22	0,20
41,00	1,00	0,22	0,16
29,00	3,00	0,21	0,20
37,00	2,00	0,18	0,19
40,00	2,00	0,14	0,13
31,00	6,00	0,25	0,15
32,00	7,00	0,19	0,16
36,00	5,00	0,21	0,19
33,00	2,00	0,27	0,20
38,00	4,00	0,16	0,15
32,00	3,00	0,14	0,12
39,00	12,00	0,19	0,16
34,00	1,00	0,34	0,22
30,00	2,00	0,19	0,16
27,00	0,00	0,27	0,29
41,00	1,00	0,24	0,18
23,00	6,00	0,12	0,12
31,00	10,00	0,16	0,16
40,00	4,00	0,18	0,16
33,00	7,00	0,12	0,11
23,00	10,00	0,17	0,16
37,00	3,00	0,16	0,19
34,00	4,00	0,28	0,28
31,00	4,00	0,14	0,14
33,00	3,00	0,21	0,11
37,00	4,00	0,17	0,16
38,00	5,00	0,38	0,34
34,00	0,00	0,23	0,23
38,00	2,00	0,22	0,25
39,00	3,00	0,29	0,25
37,00	2,00	0,17	0,15
40,00	3,00	0,24	0,19

Tins_Left_PRE	Texp_Left_PRE	Tins_Texp_Right_PRE	Tins_Texp_Left_PRE	VAS_POST	MIP_POST
0,22	0,21	0,03	0,01	0,00	137,33
0,27	0,25	0,04	0,02	0,00	84,67
0,36	0,19	0,03	0,18	2,30	101,33
0,20	0,22	-0,02	-0,02	2,00	107,00
0,13	0,18	-0,01	-0,05	2,00	68,00
0,24	0,18	-0,03	0,06	0,00	86,00
0,38	0,26	0,07	0,12	2,70	138,67
0,16	0,13	0,03	0,03	#¡NULO!	#¡NULO!
0,18	0,17	0,07	0,01	2,90	105,00
0,19	0,18	0,09	0,01	0,00	95,67
0,17	0,19	0,03	-0,02	0,50	72,00
0,22	0,19	-0,17	0,04	2,00	89,00
0,27	0,12	0,04	0,15	1,50	72,67
0,19	0,17	0,01	0,03	0,70	74,33
0,19	0,19	0,05	-0,01	1,00	142,67
0,18	0,21	0,01	-0,04	4,40	48,67
0,29	0,26	0,00	0,03	0,80	92,67
0,42	0,28	0,08	0,14	4,40	109,33
0,12	0,10	0,02	0,02	2,80	101,00
0,21	0,16	0,07	0,05	3,90	94,33
0,26	0,31	0,07	-0,05	1,50	148,67
0,17	0,16	0,05	0,01	2,40	100,67
0,32	0,25	-0,05	0,07	1,50	74,00
0,48	0,33	0,04	0,16	0,70	94,67
0,26	0,19	0,00	0,07	1,70	55,00
0,25	0,23	-0,04	0,02	7,20	110,67
0,21	0,21	0,02	0,00	0,80	110,67
0,20	0,21	0,08	-0,01	7,20	125,67
0,38	0,31	-0,01	0,06	1,60	34,33
0,20	0,15	0,08	0,05	0,50	143,67
0,32	0,33	0,04	0,00	1,10	65,00
0,26	0,22	0,00	0,04	#¡NULO!	#¡NULO!
0,22	0,22	-0,06	0,00	0,60	89,67
0,18	0,15	0,01	0,03	1,10	136,67
0,27	0,14	0,09	0,13	3,20	90,33
0,29	0,27	0,04	0,01	1,80	70,67
0,28	0,30	0,01	-0,02	#¡NULO!	#¡NULO!
0,35	0,21	0,16	0,14	#¡NULO!	#¡NULO!
0,27	0,27	0,18	0,00	#¡NULO!	#¡NULO!
0,21	0,20	-0,05	0,01	1,80	47,67
0,20	0,18	0,02	0,01	1,00	66,67
0,14	0,13	0,03	0,01	3,40	76,67
0,22	0,21	0,04	0,01	0,40	53,67
0,26	0,24	-0,01	0,02	0,70	125,33
0,19	0,18	0,05	0,02	0,40	128,00
0,20	0,18	0,01	0,02	1,20	159,33
0,22	0,17	0,02	0,05	2,30	63,67
0,16	0,15	0,04	0,02	3,00	68,67
0,22	0,18	0,04	0,03	1,50	138,00

0,16	0,16	0,02	0,00	6,20	120,00
0,21	0,21	0,09	0,01	3,50	123,67
0,22	0,20	0,00	0,02	5,90	117,00
0,21	0,19	-0,01	0,01	1,70	79,33
0,22	0,21	0,01	0,01	1,60	112,67
0,31	0,28	0,02	0,03	6,20	49,00
0,21	0,18	0,05	0,03	3,30	49,33
0,23	0,17	0,06	0,06	5,30	110,33
0,24	0,22	0,04	0,02	0,00	109,33
0,25	0,20	0,01	0,05	1,50	95,00
0,19	0,17	0,06	0,02	2,70	113,33
0,24	0,22	0,01	0,02	1,90	59,00
0,23	0,24	-0,01	0,00	1,60	119,67
0,22	0,15	0,02	0,07	0,80	63,00
0,20	0,13	0,10	0,07	3,30	40,33
0,25	0,20	0,03	0,05	2,40	105,67
0,21	0,16	0,03	0,05	0,70	93,00
0,20	0,16	0,07	0,04	4,00	121,33
0,15	0,08	0,02	0,08	0,80	115,33
0,16	0,14	0,02	0,02	1,50	112,00
0,18	0,16	0,03	0,02	2,50	46,00
0,24	0,19	0,12	0,05	#¡NULO!	#¡NULO!
0,22	0,20	0,03	0,02	0,70	118,00
0,21	0,19	-0,02	0,03	7,20	56,00
0,15	0,13	0,06	0,02	0,70	160,00
0,11	0,10	0,00	0,01	7,20	67,67
0,14	0,11	0,00	0,03	0,80	109,67
0,18	0,16	0,03	0,02	0,00	72,33
0,12	0,13	0,00	-0,01	5,20	103,33
0,14	0,11	0,00	0,03	3,10	32,67
0,14	0,13	-0,03	0,01	1,80	80,00
0,29	0,26	0,00	0,02	5,80	111,00
0,13	0,09	0,01	0,04	5,90	99,33
0,15	0,12	0,11	0,03	4,00	138,33
0,14	0,10	0,01	0,03	1,40	162,00
0,22	0,17	0,04	0,04	1,20	98,33
0,22	0,18	0,00	0,04	0,00	161,33
0,30	0,23	-0,03	0,08	0,00	190,67
0,22	0,20	0,05	0,02	3,50	70,33
0,15	0,17	0,02	-0,02	0,20	93,00
0,21	0,18	0,04	0,03	6,00	138,67

MEP_POST	FVC_POST	FEV1_POST	FEV1_FVC_POST	PPT_Right_POST	PPT_Left_POST
146,33	4,24	4,23	99,81	8,57	9,33
128,67	4,70	4,11	87,33	8,90	8,77
147,00	4,47	3,72	83,22	4,13	4,17
123,00	4,23	3,84	90,79	6,03	6,07
88,33	2,73	2,71	99,34	5,70	5,63
92,67	4,66	4,17	89,44	6,67	6,50
151,67	4,99	4,89	97,97	6,70	7,77
#¡NULO!	#¡NULO!	#¡NULO!	#¡NULO!	#¡NULO!	#¡NULO!
117,00	3,02	3,00	99,35	4,37	4,53
127,00	6,24	5,19	83,18	7,90	8,00
97,33	3,18	3,16	99,44	10,00	10,00
98,67	2,78	2,21	79,33	2,43	3,10
105,00	2,72	2,55	93,74	7,03	6,63
136,33	4,49	4,49	100,00	3,83	3,70
180,67	3,19	3,18	99,65	5,33	5,73
77,33	2,69	2,36	87,70	2,20	2,37
119,67	1,82	1,80	98,57	8,47	8,20
93,67	3,46	3,05	88,49	3,57	3,60
129,67	4,99	3,86	77,46	5,43	5,53
100,33	2,72	2,63	96,54	5,27	5,47
178,33	3,69	3,21	86,81	7,67	8,80
137,33	2,57	2,37	92,43	4,93	5,40
112,33	2,95	2,74	93,08	3,10	3,63
121,00	3,10	2,80	89,71	6,67	7,33
110,33	1,42	1,41	99,42	3,70	4,10
111,33	2,41	2,40	99,55	3,13	3,20
106,67	3,09	2,71	91,00	5,60	5,97
192,67	4,62	3,76	81,50	6,87	6,60
69,00	3,07	2,99	97,16	5,03	5,47
174,33	1,68	1,68	99,59	9,60	8,03
91,00	2,92	2,86	98,27	6,50	7,43
#¡NULO!	#¡NULO!	#¡NULO!	#¡NULO!	#¡NULO!	#¡NULO!
90,67	2,91	2,55	87,83	7,07	6,33
186,00	4,71	4,13	87,84	8,90	9,17
110,33	2,35	2,34	99,55	6,73	8,10
127,00	3,60	3,37	93,67	5,27	4,80
#¡NULO!	#¡NULO!	#¡NULO!	#¡NULO!	#¡NULO!	#¡NULO!
#¡NULO!	#¡NULO!	#¡NULO!	#¡NULO!	#¡NULO!	#¡NULO!
#¡NULO!	#¡NULO!	#¡NULO!	#¡NULO!	#¡NULO!	#¡NULO!
109,00	4,47	4,36	97,51	3,93	4,03
96,00	2,70	2,34	86,61	3,93	4,77
109,67	2,23	2,22	99,45	4,03	4,33
62,00	3,34	3,23	96,78	3,50	3,33
136,00	4,10	3,89	94,75	6,83	7,37
155,00	5,58	4,68	84,01	9,90	10,00
157,67	4,79	4,57	95,32	8,17	8,30
111,00	2,37	2,35	99,43	2,53	2,10
75,33	2,39	2,25	94,09	2,90	2,87
156,67	4,70	4,14	88,10	10,00	10,00

182,00	4,34	3,82	88,02	7,67	7,63
154,67	3,23	2,89	89,43	8,00	8,07
127,00	3,00	2,93	97,32	6,03	6,43
95,67	2,69	2,57	95,99	5,87	5,30
139,67	2,91	2,55	85,54	9,60	9,83
110,33	2,17	1,80	83,55	4,40	4,60
100,00	2,51	2,21	88,11	5,80	6,10
129,33	3,59	3,07	85,63	7,90	8,67
162,00	4,35	3,94	90,90	7,30	7,30
111,33	2,90	2,44	84,12	5,20	5,23
156,67	4,35	3,61	82,97	9,03	8,07
114,00	2,93	2,62	89,35	3,57	3,50
194,00	6,30	5,23	83,40	6,47	5,90
82,67	3,05	2,62	85,97	4,30	3,67
305,33	2,56	1,77	71,73	5,73	5,07
155,00	3,31	3,24	97,94	5,67	5,97
144,00	4,15	3,19	76,86	6,10	5,30
129,67	4,41	3,93	89,20	7,10	7,30
185,33	4,10	3,71	90,62	6,77	6,63
132,33	4,21	3,93	93,64	5,47	5,63
70,00	2,08	2,04	98,09	5,63	5,80
#¡NULO!	#¡NULO!	#¡NULO!	#¡NULO!	#¡NULO!	#¡NULO!
103,33	2,99	2,98	99,80	6,10	6,63
90,33	2,99	2,55	84,93	6,77	6,83
157,33	5,54	4,15	74,91	7,97	8,77
73,00	2,28	2,27	99,79	7,03	5,57
182,00	4,43	3,58	81,00	10,00	10,00
119,00	3,36	3,01	89,68	5,97	5,97
127,00	3,06	2,62	85,46	4,47	5,23
81,00	2,78	2,24	80,69	4,30	3,37
96,33	3,04	2,64	86,08	7,87	8,17
145,00	3,35	2,73	81,40	5,57	3,87
122,00	3,57	3,51	98,38	4,57	3,73
171,33	4,09	3,76	91,96	7,23	7,53
173,33	3,69	3,62	98,11	7,30	7,67
159,67	3,53	2,95	83,57	9,90	10,00
175,00	3,08	2,98	96,98	6,67	7,50
262,67	5,57	4,28	76,72	7,60	8,00
118,67	3,35	3,09	92,77	6,27	5,47
122,67	3,85	3,32	86,31	8,37	8,13
160,33	3,73	3,29	92,77	9,83	9,67

SF12_Physical_DIRECT_SCORES_POST	SF12_Mental_DIRECT_SCORES_POST
19,00	23,00
17,00	22,00
20,00	24,00
17,00	23,00
16,00	22,00
18,00	22,00
20,00	26,00
#¡NULO!	#¡NULO!
20,00	25,00
20,00	27,00
19,00	26,00
12,00	20,00
16,00	20,00
17,00	23,00
18,00	24,00
15,00	14,00
19,00	22,00
16,00	21,00
20,00	21,00
16,00	26,00
19,00	23,00
14,00	17,00
20,00	26,00
19,00	25,00
14,00	23,00
13,00	24,00
18,00	17,00
14,00	21,00
15,00	18,00
19,00	23,00
16,00	25,00
#¡NULO!	#¡NULO!
19,00	24,00
19,00	20,00
17,00	20,00
17,00	21,00
#¡NULO!	#¡NULO!
#¡NULO!	#¡NULO!
#¡NULO!	#¡NULO!
18,00	23,00
18,00	24,00
19,00	25,00
17,00	24,00
19,00	24,00
19,00	24,00
18,00	21,00
18,00	24,00
18,00	18,00
19,00	25,00

13,00	21,00
17,00	21,00
15,00	19,00
16,00	21,00
17,00	24,00
16,00	20,00
15,00	20,00
15,00	22,00
17,00	23,00
17,00	23,00
19,00	23,00
15,00	20,00
17,00	22,00
19,00	22,00
14,00	19,00
19,00	23,00
18,00	23,00
14,00	18,00
17,00	24,00
17,00	26,00
15,00	22,00
#¡NULO!	#¡NULO!
17,00	21,00
12,00	18,00
19,00	24,00
12,00	15,00
18,00	23,00
19,00	24,00
12,00	20,00
12,00	16,00
18,00	21,00
14,00	20,00
15,00	11,00
17,00	18,00
19,00	17,00
17,00	25,00
19,00	22,00
19,00	24,00
16,00	21,00
19,00	20,00
16,00	22,00

SF12_Total_DIRECT_SCORES_POST	Rolland_Morris_POST	Tins_Right_POST	Texp_Right_POST
42,00	0,00	0,28	0,25
39,00	1,00	0,31	0,25
44,00	0,00	0,23	0,21
40,00	2,00	0,16	0,14
38,00	4,00	0,19	0,27
40,00	0,00	0,15	0,13
46,00	1,00	0,47	0,30
#¡NULO!	#¡NULO!	#¡NULO!	#¡NULO!
45,00	1,00	0,34	0,24
47,00	0,00	0,34	0,18
45,00	0,00	0,21	0,14
32,00	6,00	0,17	0,15
36,00	4,00	0,21	0,23
40,00	2,00	0,20	0,13
42,00	2,00	0,19	0,18
29,00	3,00	0,17	0,16
41,00	0,00	0,21	0,21
37,00	2,00	0,19	0,14
41,00	0,00	0,18	0,14
42,00	3,00	0,27	0,20
42,00	4,00	0,30	0,16
31,00	2,00	0,15	0,14
46,00	4,00	0,31	0,22
44,00	4,00	0,27	0,14
37,00	4,00	0,29	0,15
37,00	5,00	0,39	0,23
35,00	1,00	0,31	0,20
35,00	7,00	0,36	0,18
33,00	0,00	0,27	0,26
42,00	0,00	0,26	0,17
41,00	1,00	0,22	0,15
#¡NULO!	#¡NULO!	#¡NULO!	#¡NULO!
43,00	0,00	0,38	0,24
39,00	0,00	0,44	0,21
37,00	2,00	0,21	0,14
38,00	4,00	0,31	0,23
#¡NULO!	#¡NULO!	#¡NULO!	#¡NULO!
#¡NULO!	#¡NULO!	#¡NULO!	#¡NULO!
#¡NULO!	#¡NULO!	#¡NULO!	#¡NULO!
41,00	0,00	0,17	0,13
42,00	0,00	0,15	0,09
44,00	2,00	0,23	0,16
41,00	0,00	0,25	0,16
43,00	0,00	0,31	0,21
43,00	3,00	0,19	0,16
39,00	0,00	0,30	0,29
42,00	2,00	0,14	0,12
36,00	2,00	0,17	0,11
44,00	0,00	0,27	0,14

34,00	3,00	0,31	0,17
38,00	4,00	0,20	0,15
34,00	7,00	0,33	0,17
37,00	4,00	0,23	0,16
41,00	4,00	0,25	0,16
36,00	6,00	0,26	0,28
35,00	8,00	0,13	0,10
37,00	2,00	0,22	0,22
40,00	0,00	0,27	0,19
40,00	0,00	0,29	0,25
42,00	2,00	0,27	0,19
35,00	2,00	0,27	0,18
39,00	1,00	0,25	0,15
41,00	1,00	0,18	0,12
33,00	2,00	0,14	0,11
42,00	4,00	0,19	0,14
41,00	0,00	0,21	0,14
32,00	3,00	0,13	0,11
41,00	2,00	0,23	0,18
43,00	0,00	0,16	0,16
37,00	11,00	0,25	0,17
#¡NULO!	#¡NULO!	#¡NULO!	#¡NULO!
38,00	0,00	0,20	0,17
30,00	9,00	0,33	0,27
43,00	0,00	0,20	0,16
27,00	7,00	0,13	0,14
41,00	1,00	0,32	0,21
43,00	2,00	0,23	0,16
32,00	11,00	0,21	0,21
28,00	7,00	0,18	0,15
39,00	0,00	0,27	0,17
34,00	4,00	0,18	0,17
26,00	3,00	0,17	0,11
35,00	2,00	0,26	0,16
36,00	1,00	0,26	0,20
42,00	0,00	0,32	0,18
41,00	0,00	0,14	0,12
43,00	0,00	0,35	0,29
37,00	1,00	0,14	0,13
39,00	1,00	0,18	0,15
38,00	2,00	0,20	0,17

Tins_Left_POST	Texp_Left_POST	Tins_Texp_Right_POST	Tins_Texp_Left_POST	VAS_DIF
0,28	0,21	0,03	0,07	-5,70
0,33	0,29	0,06	0,04	-5,00
0,35	0,31	0,02	0,04	-2,90
0,22	0,20	0,02	0,02	-2,00
0,15	0,12	-0,08	0,03	-0,50
0,13	0,13	0,01	0,00	-8,80
0,45	0,24	0,17	0,21	-1,00
#¡NULO!	#¡NULO!	#¡NULO!	#¡NULO!	#¡NULO!
0,32	0,23	0,10	0,09	-3,10
0,29	0,19	0,16	0,10	-4,40
0,16	0,23	0,07	-0,08	-4,10
0,27	0,18	0,02	0,09	-0,80
0,14	0,16	-0,02	-0,01	-1,30
0,26	0,12	0,07	0,14	-6,30
0,23	0,24	0,01	0,00	-2,10
0,16	0,13	0,01	0,03	-2,40
0,32	0,27	0,01	0,05	-2,00
0,17	0,18	0,05	-0,01	0,40
0,23	0,15	0,05	0,08	-4,70
0,24	0,22	0,07	0,02	-2,60
0,19	0,18	0,14	0,01	-3,90
0,16	0,20	0,02	-0,05	-3,00
0,21	0,19	0,09	0,03	-5,70
0,18	0,12	0,14	0,07	-0,80
0,31	0,21	0,14	0,11	-4,30
0,18	0,14	0,16	0,03	3,30
0,26	0,27	0,11	-0,01	-1,80
0,31	0,15	0,18	0,17	2,40
0,31	0,18	0,01	0,13	-3,10
0,33	0,25	0,10	0,08	-1,50
0,29	0,17	0,06	0,12	-3,70
#¡NULO!	#¡NULO!	#¡NULO!	#¡NULO!	#¡NULO!
0,24	0,24	0,14	0,00	-4,70
0,34	0,24	0,23	0,11	-4,50
0,24	0,16	0,07	0,08	-1,90
0,32	0,24	0,08	0,08	-2,20
#¡NULO!	#¡NULO!	#¡NULO!	#¡NULO!	#¡NULO!
#¡NULO!	#¡NULO!	#¡NULO!	#¡NULO!	#¡NULO!
#¡NULO!	#¡NULO!	#¡NULO!	#¡NULO!	#¡NULO!
0,16	0,11	0,04	0,05	0,20
0,17	0,12	0,06	0,05	-4,30
0,21	0,14	0,07	0,07	-1,10
0,20	0,17	0,09	0,03	-5,80
0,24	0,25	0,11	-0,01	-2,50
0,21	0,19	0,03	0,02	-5,60
0,24	0,21	0,01	0,03	-1,10
0,16	0,14	0,02	0,02	-4,40
0,15	0,14	0,06	0,02	-5,20
0,22	0,24	0,13	-0,02	-3,40

0,32	0,21	0,14	0,12	-0,40
0,15	0,14	0,05	0,01	-0,60
0,20	0,14	0,15	0,06	-2,80
0,24	0,18	0,07	0,07	-1,60
0,16	0,13	0,09	0,04	-2,50
0,24	0,21	-0,02	0,03	1,20
0,13	0,13	0,03	0,00	-3,00
0,18	0,18	0,01	0,00	-1,00
0,19	0,17	0,08	0,02	-2,90
0,35	0,32	0,04	0,03	-1,50
0,19	0,20	0,09	-0,01	-0,30
0,24	0,17	0,09	0,08	0,10
0,21	0,20	0,10	0,01	-2,20
0,16	0,12	0,05	0,04	-3,10
0,13	0,12	0,03	0,01	-0,40
0,22	0,16	0,05	0,06	-2,70
0,22	0,21	0,08	0,02	-4,40
0,20	0,17	0,02	0,03	-1,50
0,20	0,15	0,05	0,05	-4,90
0,21	0,17	-0,01	0,03	-2,50
0,27	0,16	0,08	0,11	-4,70
#¡NULO!	#¡NULO!	#¡NULO!	#¡NULO!	#¡NULO!
0,32	0,23	0,03	0,09	-2,40
0,35	0,35	0,06	0,00	-0,20
0,21	0,19	0,04	0,01	-0,80
0,14	0,12	-0,01	0,03	-0,30
0,22	0,19	0,12	0,02	-6,10
0,24	0,19	0,07	0,05	-4,50
0,23	0,16	-0,01	0,08	-0,90
0,18	0,14	0,02	0,04	-3,80
0,22	0,15	0,09	0,07	-6,40
0,25	0,19	0,02	0,06	1,50
0,12	0,10	0,05	0,02	0,90
0,23	0,19	0,11	0,05	-0,90
0,26	0,17	0,06	0,10	-4,60
0,30	0,26	0,14	0,04	-1,10
0,18	0,18	0,02	0,00	-3,50
0,43	0,28	0,06	0,16	-7,00
0,13	0,13	0,01	0,00	-0,40
0,16	0,14	0,04	0,03	-2,20
0,23	0,18	0,03	0,05	-0,40

MIP_DIF	MEP_DIF	FVC_DIF	FEV1_DIF	FEV1_FVC_DIF	PPT_Right_DIF	PPT_Left_DIF
53,00	40,00	0,29	0,29	-0,04	3,30	4,83
26,00	60,34	0,24	0,25	0,65	1,50	0,64
58,33	81,67	0,04	-0,01	-0,88	1,40	1,57
59,00	39,33	0,03	0,08	1,25	1,46	1,27
29,33	32,33	0,40	0,39	-0,13	0,90	0,76
14,67	-19,66	0,50	0,06	-9,38	0,94	0,40
51,34	39,34	0,19	0,12	-1,33	1,37	2,07
#¡NULO!	#¡NULO!	#¡NULO!	#¡NULO!	#¡NULO!	#¡NULO!	#¡NULO!
64,67	41,00	0,28	0,26	-0,59	-1,40	-1,40
15,67	-18,33	0,21	0,26	1,51	3,97	3,93
13,00	13,00	-0,32	-0,30	0,54	5,73	5,73
26,67	28,34	0,44	-0,06	-17,86	0,86	1,40
27,34	2,00	0,42	0,26	-5,67	0,70	-0,17
10,00	-17,00	0,59	0,59	0,06	1,16	0,87
56,67	50,00	0,21	0,23	0,40	1,10	1,53
17,34	26,00	0,26	0,11	-4,87	-0,07	0,04
7,00	7,67	-0,38	-0,40	-1,22	3,07	2,53
46,66	29,67	0,12	-0,13	-6,76	-0,23	0,40
24,67	12,00	1,49	1,21	1,66	1,13	1,90
5,66	3,66	-0,06	0,00	1,84	0,27	0,77
46,67	57,00	0,08	0,09	0,44	3,30	4,30
20,34	-13,00	-0,04	0,10	5,30	1,23	1,33
13,67	33,33	0,44	0,32	-3,23	0,70	0,63
54,34	70,00	0,01	-0,05	-2,50	1,54	2,10
12,00	36,00	0,03	0,04	0,42	1,77	1,53
50,67	28,00	-0,68	-0,66	0,42	1,23	1,23
24,00	30,34	-0,08	-0,07	3,56	1,40	1,94
43,67	38,34	0,16	0,11	-1,06	-1,46	-2,07
4,66	29,00	0,56	0,48	-2,67	2,20	1,80
74,00	59,66	0,19	0,19	0,14	3,60	1,63
2,67	12,33	0,19	0,18	0,08	1,27	3,96
#¡NULO!	#¡NULO!	#¡NULO!	#¡NULO!	#¡NULO!	#¡NULO!	#¡NULO!
28,34	30,67	0,83	0,52	-10,21	2,94	2,40
18,00	0,33	0,24	-0,01	-4,86	3,73	4,17
48,66	49,66	-0,67	-0,46	6,76	1,00	1,77
11,00	3,67	-0,26	-0,32	-1,86	1,57	1,80
#¡NULO!	#¡NULO!	#¡NULO!	#¡NULO!	#¡NULO!	#¡NULO!	#¡NULO!
#¡NULO!	#¡NULO!	#¡NULO!	#¡NULO!	#¡NULO!	#¡NULO!	#¡NULO!
#¡NULO!	#¡NULO!	#¡NULO!	#¡NULO!	#¡NULO!	#¡NULO!	#¡NULO!
7,67	4,00	0,51	0,76	5,18	-0,17	-0,27
4,34	18,67	0,33	0,09	-8,30	1,13	2,17
24,00	38,67	0,33	0,34	0,41	1,93	1,60
36,00	34,67	0,08	0,11	1,24	2,10	2,03
69,66	83,33	0,56	0,35	-5,19	0,93	1,47
21,00	-7,00	1,49	0,63	-15,00	3,87	4,87
11,66	-13,33	0,87	0,66	-4,61	2,24	2,83
20,67	0,00	0,38	0,36	-0,49	0,60	0,60
31,67	18,66	-0,18	-0,09	2,89	-0,73	-0,16
39,33	28,34	1,06	0,51	-11,65	3,07	3,13

21,67	0,67	0,39	0,00	-8,88	2,00	2,30
32,67	51,34	0,51	0,21	-9,44	3,33	3,54
70,33	50,33	-0,32	-0,06	7,24	4,43	4,53
12,33	9,67	0,64	0,53	-3,43	3,07	2,67
8,34	-27,33	0,42	0,24	-7,17	2,13	1,10
13,67	34,66	0,16	0,08	-2,34	1,70	1,80
14,33	21,33	-0,68	-0,32	8,56	2,07	1,90
10,00	17,00	-0,06	-0,45	-1,95	2,47	4,14
-3,00	9,67	2,48	2,07	-8,86	0,90	1,10
35,67	30,33	1,21	0,76	-15,31	-0,37	1,73
41,66	5,34	-0,46	-0,33	1,10	2,20	1,24
22,67	46,00	-0,57	-0,57	-1,16	-0,80	-0,40
11,67	41,67	0,84	0,63	-0,63	2,37	1,97
15,00	24,34	0,35	-0,02	2,19	-0,30	-0,83
4,66	260,33	0,86	0,08	-27,62	2,83	1,70
13,00	40,67	0,82	0,83	0,93	2,70	2,74
59,33	81,67	0,50	-0,31	-19,28	3,30	2,63
66,33	14,34	0,41	0,30	-1,64	-1,77	-1,70
44,33	56,33	0,26	0,14	-2,40	3,87	3,53
26,00	7,00	1,15	0,88	-6,06	-1,00	-0,17
12,33	25,00	0,15	0,20	2,62	0,53	0,53
#¡NULO!	#¡NULO!	#¡NULO!	#¡NULO!	#¡NULO!	#¡NULO!	#¡NULO!
44,00	20,00	0,49	0,50	0,94	1,67	2,46
15,00	7,66	0,19	-0,07	-8,40	1,70	1,53
27,00	-1,00	2,26	0,92	-23,64	2,14	1,90
23,00	35,33	0,67	0,70	2,16	2,93	1,77
38,00	20,67	-0,10	-0,25	-3,48	2,87	2,90
17,33	36,00	-0,01	-0,06	-1,40	1,07	1,27
45,33	26,33	-0,06	-0,10	-1,49	1,87	2,60
-4,33	16,67	-0,08	-0,30	-8,19	2,30	1,34
32,67	16,00	-0,11	-0,15	-2,41	2,37	2,00
26,33	36,33	-0,16	-0,42	-8,57	1,40	-0,20
35,00	52,67	0,47	0,42	-1,15	2,04	1,70
20,00	8,66	-0,14	-0,33	-4,57	2,40	3,46
19,33	8,66	-0,13	-0,18	-1,15	2,57	3,70
-4,00	-8,66	0,42	-0,05	-13,01	6,03	6,50
8,00	12,67	-0,11	0,02	3,95	-0,20	0,10
53,34	88,00	1,44	0,19	-22,29	1,47	2,27
13,00	24,00	-0,15	-0,15	0,12	-0,43	-0,50
8,00	2,00	0,03	-0,01	-0,91	-1,63	-1,77
26,34	0,66	0,00	-0,15	0,66	0,50	-0,26

SF12_Physical_DIRECT_SCORES_DIF	SF12_Mental_DIRECT_SCORES_DIF
5,00	-1,00
1,00	1,00
4,00	5,00
0,00	3,00
0,00	3,00
2,00	1,00
2,00	1,00
#¡NULO!	#¡NULO!
2,00	4,00
5,00	3,00
3,00	6,00
-4,00	-2,00
1,00	2,00
1,00	0,00
2,00	7,00
2,00	-3,00
1,00	-1,00
-2,00	1,00
1,00	-1,00
4,00	7,00
5,00	2,00
0,00	-2,00
2,00	0,00
1,00	3,00
0,00	2,00
-1,00	3,00
4,00	0,00
-3,00	1,00
2,00	0,00
2,00	2,00
-1,00	1,00
#¡NULO!	#¡NULO!
4,00	3,00
2,00	2,00
2,00	2,00
-1,00	0,00
#¡NULO!	#¡NULO!
#¡NULO!	#¡NULO!
#¡NULO!	#¡NULO!
1,00	0,00
5,00	4,00
1,00	0,00
3,00	4,00
3,00	2,00
5,00	4,00
3,00	1,00
5,00	1,00
4,00	1,00
0,00	3,00

	-1,00		1,00
	2,00		-1,00
	1,00		0,00
	-1,00		-3,00
	1,00		3,00
	1,00		-1,00
	0,00		2,00
	3,00		4,00
	1,00		3,00
	1,00		2,00
	1,00		0,00
	0,00		6,00
	0,00		2,00
	2,00		-1,00
	-1,00		3,00
	7,00		3,00
	1,00		4,00
	0,00		-1,00
	0,00		3,00
	1,00		10,00
	0,00		-2,00
#¡NULO!		#¡NULO!	
	2,00		6,00
	1,00		2,00
	0,00		2,00
	2,00		2,00
	5,00		5,00
	2,00		1,00
	-1,00		0,00
	1,00		4,00
	1,00		1,00
	3,00		-3,00
	-2,00		-3,00
	1,00		1,00
	1,00		-2,00
	2,00		2,00
	1,00		6,00
	1,00		4,00
	-2,00		0,00
	2,00		0,00
	0,00		-2,00

SF12_Total_DIRECT_SCORES_DIF	Rolland_Morris_DIF	Tins_Right_DIF	Texp_Right_DIF
4,00	-11,00	0,07	0,06
2,00	-2,00	0,11	0,09
9,00	0,00	-0,08	-0,06
3,00	0,00	0,03	-0,01
3,00	-3,00	0,05	0,12
3,00	0,00	-0,08	-0,12
3,00	-1,00	0,09	-0,01
#¡NULO!	#¡NULO!	#¡NULO!	#¡NULO!
6,00	-2,00	0,12	0,08
8,00	-4,00	0,06	-0,01
9,00	-3,00	0,00	-0,05
-6,00	1,00	0,17	-0,03
3,00	-8,00	-0,01	0,05
1,00	0,00	0,08	0,02
9,00	-2,00	-0,05	-0,02
-1,00	-7,00	0,00	0,00
0,00	-3,00	0,02	0,01
-1,00	0,00	-0,15	-0,12
2,00	-2,00	-0,01	-0,03
11,00	-7,00	-0,03	-0,03
7,00	-7,00	0,02	-0,06
-2,00	0,00	-0,02	0,01
2,00	-1,00	0,08	-0,06
4,00	-1,00	0,09	-0,01
2,00	0,00	0,10	-0,04
2,00	2,00	0,20	0,00
4,00	-3,00	0,15	0,05
-2,00	2,00	0,11	0,00
2,00	-5,00	0,06	0,04
4,00	-1,00	0,03	0,01
0,00	-2,00	-0,02	-0,04
#¡NULO!	#¡NULO!	#¡NULO!	#¡NULO!
7,00	-2,00	0,09	-0,11
4,00	-6,00	0,27	0,05
4,00	-1,00	-0,09	-0,07
-1,00	-5,00	0,05	0,01
#¡NULO!	#¡NULO!	#¡NULO!	#¡NULO!
#¡NULO!	#¡NULO!	#¡NULO!	#¡NULO!
#¡NULO!	#¡NULO!	#¡NULO!	#¡NULO!
1,00	-3,00	0,01	-0,07
9,00	-2,00	0,02	-0,03
1,00	0,00	0,04	-0,01
7,00	-2,00	0,05	0,00
5,00	-2,00	0,10	-0,01
9,00	-8,00	-0,06	-0,04
4,00	-3,00	-0,01	-0,01
6,00	-3,00	-0,03	-0,03
5,00	-2,00	0,02	-0,01
3,00	-1,00	0,03	-0,06

0,00	0,00	0,06	-0,06
1,00	-2,00	-0,10	-0,06
1,00	1,00	0,07	-0,08
-4,00	-1,00	0,05	-0,03
4,00	0,00	0,10	0,03
0,00	2,00	0,09	0,12
2,00	-4,00	-0,08	-0,06
7,00	-2,00	0,02	0,08
4,00	-1,00	0,06	0,03
3,00	-3,00	0,07	0,04
1,00	1,00	0,06	0,02
6,00	-1,00	0,06	-0,02
2,00	-1,00	0,07	-0,04
1,00	-1,00	0,03	0,00
2,00	-4,00	-0,12	-0,04
10,00	-3,00	0,00	-0,02
5,00	-5,00	0,00	-0,05
-1,00	1,00	-0,14	-0,09
3,00	-2,00	0,07	0,03
11,00	-3,00	0,02	0,04
-2,00	-1,00	0,06	0,01
#¡NULO!	#¡NULO!	#¡NULO!	#¡NULO!
8,00	-2,00	0,01	0,01
3,00	9,00	0,06	-0,02
2,00	-1,00	-0,04	-0,03
4,00	1,00	0,01	0,02
10,00	-9,00	0,16	0,05
3,00	-2,00	0,05	0,01
-1,00	4,00	0,09	0,10
5,00	-3,00	0,01	-0,01
2,00	-3,00	0,11	-0,01
0,00	0,00	-0,10	-0,11
-5,00	-1,00	0,03	-0,02
2,00	-1,00	0,05	0,05
-1,00	-3,00	0,10	0,05
4,00	-5,00	-0,05	-0,16
7,00	0,00	-0,09	-0,11
5,00	-2,00	0,13	0,04
-2,00	-2,00	-0,15	-0,11
2,00	-1,00	0,01	-0,01
-2,00	-1,00	-0,04	-0,03

Tins_Left_DIF	Texp_Left_DIF	Tins_Texp_Right_DIF	Tins_Texp_Left_DIF
0,06	0,00	0,00	0,06
0,06	0,05	0,02	0,02
-0,01	0,13	-0,01	-0,14
0,02	-0,02	0,05	0,04
0,02	-0,06	-0,07	0,08
-0,11	-0,05	0,04	-0,06
0,07	-0,02	0,10	0,09
#¡NULO!	#¡NULO!	#¡NULO!	#¡NULO!
0,14	0,06	0,04	0,08
0,10	0,01	0,07	0,09
-0,01	0,05	0,05	-0,06
0,05	-0,01	0,19	0,06
-0,12	0,03	-0,06	-0,16
0,06	-0,05	0,07	0,11
0,05	0,05	-0,04	0,00
-0,02	-0,09	0,00	0,07
0,03	0,01	0,01	0,02
-0,25	-0,10	-0,03	-0,15
0,11	0,05	0,03	0,07
0,03	0,06	0,00	-0,03
-0,07	-0,12	0,08	0,05
-0,01	0,05	-0,03	-0,06
-0,11	-0,07	0,14	-0,04
-0,30	-0,21	0,10	-0,09
0,06	0,02	0,14	0,04
-0,07	-0,09	0,20	0,01
0,05	0,06	0,09	-0,01
0,12	-0,06	0,10	0,17
-0,06	-0,13	0,02	0,07
0,13	0,09	0,02	0,04
-0,03	-0,16	0,02	0,12
#¡NULO!	#¡NULO!	#¡NULO!	#¡NULO!
0,02	0,02	0,20	0,00
0,17	0,09	0,22	0,08
-0,03	0,01	-0,02	-0,04
0,03	-0,03	0,04	0,06
#¡NULO!	#¡NULO!	#¡NULO!	#¡NULO!
#¡NULO!	#¡NULO!	#¡NULO!	#¡NULO!
#¡NULO!	#¡NULO!	#¡NULO!	#¡NULO!
-0,05	-0,09	0,08	0,04
-0,03	-0,06	0,04	0,03
0,07	0,02	0,04	0,06
-0,02	-0,05	0,05	0,02
-0,02	0,00	0,11	-0,03
0,01	0,01	-0,02	0,00
0,04	0,03	0,00	0,01
-0,06	-0,03	0,00	-0,03
-0,01	-0,01	0,02	0,00
0,01	0,06	0,09	-0,05

0,17	0,05	0,12	0,12
-0,06	-0,07	-0,04	0,01
-0,02	-0,06	0,15	0,04
0,04	-0,02	0,08	0,05
-0,06	-0,09	0,08	0,02
-0,07	-0,07	-0,04	0,00
-0,08	-0,05	-0,02	-0,03
-0,05	0,01	-0,06	-0,06
-0,05	-0,05	0,04	0,00
0,10	0,12	0,03	-0,02
0,00	0,02	0,03	-0,02
0,00	-0,06	0,09	0,06
-0,03	-0,04	0,11	0,01
-0,06	-0,03	0,04	-0,03
-0,07	-0,01	-0,08	-0,06
-0,02	-0,04	0,02	0,01
0,01	0,04	0,05	-0,03
0,00	0,01	-0,05	-0,01
0,04	0,07	0,04	-0,03
0,05	0,04	-0,02	0,02
0,08	0,00	0,05	0,08
#¡NULO!	#¡NULO!	#¡NULO!	#¡NULO!
0,10	0,03	0,00	0,07
0,14	0,16	0,08	-0,03
0,05	0,06	-0,02	-0,01
0,03	0,02	-0,01	0,01
0,08	0,08	0,12	0,00
0,05	0,02	0,04	0,03
0,11	0,03	-0,01	0,08
0,04	0,03	0,02	0,01
0,08	0,02	0,12	0,06
-0,03	-0,07	0,01	0,04
-0,01	0,01	0,05	-0,02
0,08	0,07	0,00	0,02
0,12	0,06	0,05	0,06
0,08	0,09	0,11	-0,01
-0,04	0,00	0,02	-0,04
0,13	0,05	0,09	0,08
-0,09	-0,07	-0,04	-0,02
0,01	-0,04	0,02	0,05
0,02	0,00	-0,02	0,03
